# Budget-Prioritized Dynamic Regrouping for Edge Federated Services Under Workload Drift and Privacy-Budget Constraints

**DOI:** 10.3390/e28091000

**Published:** 2026-09-07

**Authors:** Li Zhao, Long Chen, Zhongyi Chen

**Affiliations:** School of Computer Science and Engineering, Southeast University, Nanjing 211189, China; lizhaolz@seu.edu.cn (L.Z.); alphonsochen@seu.edu.cn (Z.C.)

**Keywords:** federated learning, edge federated services, dynamic regrouping, privacy budget, workload drift, migration or reconfiguration cost

## Abstract

Federated learning enables model training in edge and distributed service environments without directly sharing raw data. In long-running edge federated services, fixed collaboration structures may become inefficient under workload drift, whereas frequent regrouping can incur migration overhead, group churn, and additional privacy-budget consumption. This paper studies budget-prioritized dynamic regrouping under workload drift, privacy-budget constraints, and migration or reconfiguration cost. We formulate a dynamic regrouping problem that jointly captures workload pressure, remaining privacy budget, service utility, and regrouping cost. We propose Budget-Prioritized Dynamic Regrouping (BP-DR), a triggered local method that evaluates single-node candidate operations and commits at most one regrouping operation per time slot. A candidate is accepted only when it is privacy-budget feasible and its utility improvement exceeds a threshold combining an anti-oscillation margin, migration or reconfiguration cost, and privacy-budget opportunity cost. We derive this trigger from a one-step local comparison and establish its monotonicity with respect to migration or reconfiguration cost and remaining privacy budget. Trace-driven experiments based on Alibaba Cluster Trace 2018 show that BP-DR maintains competitive migration-adjusted utility while controlling regrouping activity across dynamic and stress-test settings. FLamby Fed-Heart-Disease validation further shows similar learning performance across the compared methods while demonstrating the integration of BP-DR with group-aware federated training.

## 1. Introduction

Federated learning has become an important collaborative learning paradigm for edge and distributed service environments, enabling multiple institutions, service providers, or edge nodes to jointly train models without directly sharing raw data [1,2]. In practical edge federated services, collaboration structures may evolve with service demand, workload intensity, communication pressure, and client states, while participating nodes may have heterogeneous privacy sensitivity and limited privacy budgets. Recent studies have explored dynamic grouping, privacy-budget adjustment, clustered federated learning, and dynamic client collaboration under heterogeneous or drifting conditions [3,4,5,6]. Nevertheless, static grouping may become inefficient under workload drift, whereas frequent regrouping can introduce migration overhead, group churn, and additional privacy-budget consumption. Existing studies therefore provide limited guidance on when grouping should be updated under the joint effects of workload drift, remaining privacy budget, and migration or reconfiguration cost.

In this paper, we study budget-prioritized dynamic regrouping for edge federated services under these coupled conditions. The grouping structure evolves over discrete time slots, and each node has time-varying workload, budget, grouping, service-quality, and learning states. A candidate operation may improve immediate utility by moving a node to a more suitable group, but may also consume additional privacy budget and incur reconfiguration cost. The objective is therefore to balance service utility, migration overhead, and privacy-budget sustainability over time.

This problem is challenging for three reasons. First, workload drift changes group utilities over time and may make a previously suitable grouping less effective. Second, privacy budget is a finite continuation resource, so current expenditure may reduce future participation capability when the remaining budget becomes scarce. Third, aggressive reactions to short-term workload fluctuations may accumulate migration and reconfiguration costs. Consequently, periodic regrouping may perform unnecessary updates, while utility-only triggering may over-regroup and degrade the migration-adjusted objective.

To address these challenges, we propose Budget-Prioritized Dynamic Regrouping (BP-DR), a triggered local regrouping method for edge federated services. Unlike existing event-triggered regrouping approaches that mainly consider workload variations or utility improvements, and budget-aware approaches that primarily focus on privacy expenditure allocation, BP-DR formulates regrouping as a budget-prioritized trigger decision problem. Specifically, BP-DR jointly considers immediate utility improvement, migration or reconfiguration overhead, and privacy-budget opportunity cost, where the remaining privacy budget is treated as a continuation resource rather than only a consumable quantity. BP-DR evaluates single-node candidate operations and commits at most one regrouping operation per time slot. A candidate is accepted only when it is privacy-budget feasible and its immediate utility improvement exceeds a threshold combining a base anti-oscillation margin, migration or reconfiguration cost, and privacy-budget opportunity cost. The main contributions are summarized as follows.

(i) We formulate a dynamic regrouping problem for edge federated services that jointly captures workload drift, remaining privacy budget, migration or reconfiguration cost, and service-quality-related utility.
(ii) We propose BP-DR, a budget-prioritized trigger mechanism that treats the remaining privacy budget as a continuation resource and suppresses unnecessary regrouping through migration-aware and budget-aware triggering.
(iii) We derive the threshold-form trigger from a one-step local regrouping comparison and show that it becomes more conservative as migration cost increases or the remaining privacy budget tightens.
(iv) We conduct trace-driven experiments based on Alibaba Cluster Trace 2018, covering the main comparison, workload-drift robustness, migration and churn stress, ablation, privacy-budget scarcity, and scale sensitivity. The results characterize BP-DR as a tradeoff-oriented method that retains competitive migration-adjusted utility with controlled regrouping activity. An FLamby Fed-Heart-Disease validation further provides an integration test in which pooled test accuracy and loss remain similar across the compared methods while BP-DR retains its regrouping capability.

The remainder of this paper is organized as follows. Section 2 reviews related work. Section 3 presents the system model and problem formulation. Section 4 introduces BP-DR, and Section 5 analyzes its budget-prioritized trigger. Section 6 reports the experimental results, and Section 7 concludes the paper.

## 2. Related Work

Federated learning has been widely studied as a collaborative learning paradigm for edge and distributed service environments, enabling multiple participants to train models without directly sharing raw data. Existing surveys summarize its architectures, challenges, and applications in edge computing, including communication efficiency, resource management, privacy protection, and system heterogeneity [1,2]. Recent research has increasingly moved from static federation structures toward adaptive grouping, clustered learning, and dynamic client collaboration. However, when regrouping should be triggered under the joint effects of workload drift, privacy-budget constraints, and migration or reconfiguration cost remains insufficiently explored.

One research stream focuses on dynamic grouping and clustered federated learning. DGDPFL considers dynamic grouping together with privacy-budget adjustment in networked service management [3], while TDCFL addresses time-varying client distributions through adaptive similarity computation [4]. FedPLC studies dynamic cluster adaptation under concept drift and non-IID data [5], and DecantFed jointly considers client clustering, bandwidth allocation, and local training in semi-synchronous federated learning [6]. Other clustered or adaptive methods improve personalization, communication efficiency, or robustness under non-IID and imbalanced data through client clustering or selection [7,8,9,10,11,12,13]. Game-theoretic and preference-based formulations have also been used to model participant-level grouping and continual cooperation [14,15,16,17]. Although these studies demonstrate the value of adaptive collaboration, their decisions are mainly driven by similarity, clustering quality, concept drift, communication efficiency, or personalization. They generally do not model the remaining privacy budget as a continuation resource whose opportunity cost directly affects whether regrouping should be triggered.

Another body of work investigates privacy-budget-aware differentially private federated learning. Kiani et al. study time-adaptive privacy spending across training rounds [18], AWE-DPFL combines adaptive weighting with dynamic budget allocation for heterogeneous IoT data [19], and Chen et al. use multi-agent reinforcement learning for adaptive privacy-budget allocation [20]. Other methods consider adaptive differential privacy, fairness-aware client selection, dynamic budget allocation, and privacy-budget-aware ensemble learning [21,22,23,24]. These studies show that privacy budgets should not be treated as fixed or uniformly consumed resources. However, they mainly address how budgets are allocated during training rather than how the remaining budget should influence structural regrouping decisions. A regrouping-oriented perspective should instead account for the opportunity cost of consuming budget when deciding whether an update is worthwhile.

Migration, switching, and reconfiguration costs have also been considered in federated learning and edge intelligence. Hermes studies multi-job federated learning with switching cost in wireless networks [25], while FedCM investigates client clustering and migration using gradient-path similarity and update-direction deviation [26]. Cost-efficient federated learning and sequential model migration have been studied in multi-cell and serverless edge environments [27,28], and broader migration and orchestration studies show that frequent reconfiguration can introduce non-negligible operational overhead [29,30,31]. These works motivate migration-aware decision-making, but typically treat migration or switching cost as an operational penalty rather than combining it with privacy-budget opportunity cost in a regrouping trigger.

Event-triggered control provides a related principle for distributed and networked systems. Updates, communication, or actions are performed only when a measured deviation exceeds a threshold, thereby balancing responsiveness and stability while suppressing unnecessary overhead [32]. Although developed mainly for general control and optimization settings, this anti-oscillation principle is also relevant to dynamic regrouping, where small fluctuations should not cause frequent structural changes.

Workload dynamics are another important consideration in edge federated services. Workload intensity, service demand, and resource pressure may change over time and affect participation, delay, and collaborative learning performance. Existing studies address dynamic workload balancing in fog networks, federated scheduling for edge–cloud resource allocation, and federated learning under evolving distribution shifts [33,34,35]. These studies establish workload drift as a realistic system condition, but generally examine scheduling, balancing, or distribution adaptation rather than combining workload dynamics with privacy-budget constraints and reconfiguration cost in a regrouping trigger.

Overall, prior work has studied dynamic grouping, clustered federated learning, adaptive privacy-budget allocation, migration-aware collaboration, event-triggered updates, and workload-aware edge services largely as separate problems. Few studies explicitly treat the remaining privacy budget as a continuation resource whose opportunity cost affects structural updates. Moreover, to the best of our knowledge, no existing work jointly incorporates workload drift, privacy-budget constraints, and migration or reconfiguration cost into a single triggering mechanism for dynamic regrouping in edge federated services. This gap motivates BP-DR, which jointly considers migration cost, privacy-budget opportunity cost, and an anti-oscillation margin when determining whether local regrouping should be triggered. Table 1 summarizes the comparison with representative works.

## 3. System Model and Problem Formulation

This section presents the system model and formulates the dynamic regrouping problem for edge federated services under workload drift, privacy-budget constraints, and reconfiguration cost. The objective is to determine when and under what constraints an existing grouping structure should be updated while avoiding excessive migration overhead and unstable collaboration. The main notations used in the model and the proposed method are summarized in Table 2.

### 3.1. Dynamic Edge Federated Service Model

**Figure 1:**
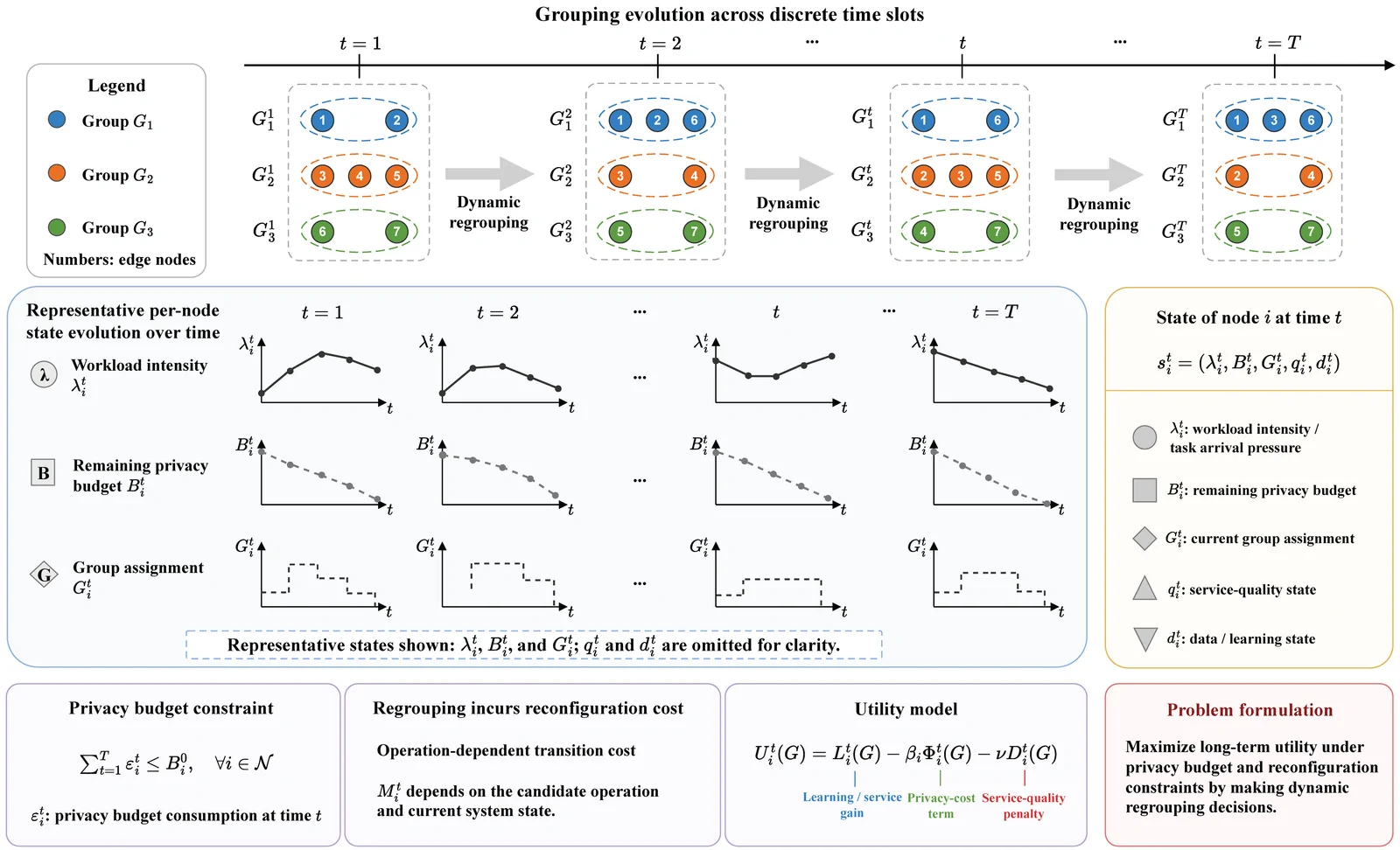
Overview of the system model for dynamic regrouping in edge federated services across discrete time slots. Numbers inside the circles denote edge-node identifiers, while the colored dashed outlines indicate group membership.

We consider a long-running edge federated service system in which participating nodes collaborate over discrete time slots. As illustrated in Figure 1, the grouping structure and node states evolve over time under privacy-budget and reconfiguration-cost constraints. In the federated-learning instantiation considered in this paper, each group corresponds to a set of nodes that jointly perform federated model updates or service-oriented federated training, and regrouping changes the collaboration set used in subsequent update windows.

Consider a set of participating nodes N={1,2,…,N}, and let the system evolve over discrete time slots t=1,2,…,T. At each time slot, nodes collaborate by forming groups for federated training and service coordination. Let

(1) $$\Pi^{t}=G_{1}^{t},G_{2}^{t},\ldots ,G_{K_{t}}^{t}$$

denote the grouping structure at time *t*, where $G_{k}^{t}\subseteq N$ is the *k*-th group and $K_{t}$ is the number of groups. The grouping structure satisfies

(2) $$\overset{K_{t}}{\underset{k=1}{⋃}}G_{k}^{t}=N,\;G_{k}^{t}\cap G_{l}^{t}=\emptyset ,\;\forall k\neq l.$$

The initial grouping structure $\Pi^{1}$ is assumed to be obtained by a standard static initialization method, such as similarity-based grouping using initial data, workload, or service-state summaries. The focus of this paper is not on initial grouping construction, but on when and how an existing grouping structure should be updated over time.

For each node *i*, $G_{i}^{t}$ denotes its group at time *t*.

Each node has time-varying service, learning, and budget states. The state of node *i* at time *t* is represented as

(3) $$s_{i}^{t}=\left(\lambda_{i}^{t},B_{i}^{t},G_{i}^{t},q_{i}^{t},d_{i}^{t}\right),$$

where the components, respectively, represent workload intensity, remaining privacy budget, current group assignment, service-quality state, and learning-related state. Their concrete instantiations may vary across experimental settings, but all state variables are assumed to be observable or estimable before a regrouping decision is made.

Workload dynamics are captured by the time-varying workload intensity $\lambda_{i}^{t}$, which affects both the utility of staying in the current group and the potential gain of moving to another group. We treat workload dynamics as an external driver of utility variation rather than as an independent modeling contribution.

### 3.2. Candidate Regrouping Model

The grouping structure may be updated over time through local regrouping operations. Instead of recomputing a global grouping structure at every time slot, the system evaluates candidate local operations. A candidate operation is restricted to a single-node local operation, such as moving from the current group to another existing group or leaving the current group to form a singleton group. Multi-node operations such as swaps or split–merge adjustments are not explicitly modeled in the core decision rule; they may be approximated by a sequence of accepted single-node operations over multiple time slots.

We denote a candidate regrouping operation for node *i* at time *t* by $a_{i}^{t}$, and the candidate group after applying this operation by $G_{i}^{t,\mathrm{new}}$. The immediate utility improvement brought by this candidate operation is defined as

(4) $$\Delta U_{i}^{t}=U_{i}^{t}\left(G_{i}^{t,\mathrm{new}}\right)-U_{i}^{t}\left(G_{i}^{t}\right),$$

where $U_{i}^{t}\left(G\right)$ denotes the immediate utility of node *i* when it belongs to group *G* at time *t*. A positive $\Delta U_{i}^{t}$ indicates an immediate utility gain from the candidate operation.

However, a positive immediate utility improvement alone is not sufficient to justify regrouping. A regrouping operation may consume additional privacy budget, incur migration or reconfiguration cost, and increase group churn. Therefore, the regrouping decision must be evaluated under both budget feasibility and reconfiguration cost constraints.

### 3.3. Privacy Budget and Reconfiguration Cost

Each node is associated with an initial privacy budget $B_{i}^{0}$. During the dynamic federated service process, node *i* consumes privacy budget when participating in federated training or group updates under the decision-level privacy-resource accounting model. Let $\varepsilon_{i}^{t}$ denote the privacy budget consumed by node *i* at time *t*. The cumulative privacy consumption is constrained by

(5) $$\sum_{t=1}^{T}\varepsilon_{i}^{t}\leq B_{i}^{0},\;\forall i\in N.$$

Equivalently, the remaining privacy budget evolves as

(6) $$B_{i}^{t+1}=B_{i}^{t}-\varepsilon_{i}^{t}.$$

For a candidate regrouping operation, let $\Delta \varepsilon_{i}^{t}$ denote a conservative estimate of the additional decision-level privacy expenditure associated with accepting the operation and entering the subsequent update window. When the operation is accepted, the realized privacy consumption is charged to $\varepsilon_{i}^{t}$ according to the same decision-level accounting rule.

In the present work, $B_{i}^{t}$ and $\Delta \varepsilon_{i}^{t}$ are used as decision-level privacy-resource accounting variables for regrouping. Accordingly, $\Delta \varepsilon_{i}^{t}$ is not the output of a formal differential-privacy accountant, and the resulting accounting does not by itself establish an end-to-end composed differential-privacy guarantee across federated-learning rounds. Throughout the remainder of this paper, the terms “privacy budget,” “privacy expenditure,” and “budget consumption” refer to this decision-level privacy-resource accounting model unless explicitly stated otherwise. Integrating the regrouping layer with a formal privacy accountant is left for future work.

Before a candidate operation can be accepted, it must satisfy the privacy-budget feasibility condition

(7) $$B_{i}^{t}-\Delta \varepsilon_{i}^{t}\geq B_{i}^{\min}.$$

Here, $B_{i}^{\min}\geq 0$ is a reserved lower bound on the remaining privacy budget; it can be set to zero or kept positive when future mandatory updates require reserved budget. If this condition is violated, the candidate regrouping operation is discarded.

A candidate regrouping operation also incurs an operation-dependent migration or reconfiguration cost proxy, denoted by $M_{i}^{t}$. In the trace-driven experiments, this proxy is instantiated as a scaled count of group-assignment changes induced by the candidate operation. In real deployments, the same term may correspond to communication overhead, model transfer, service handoff, or temporary coordination overhead. Frequent regrouping may therefore reduce system stability even when some local utility improvement is observed.

To capture the future value of preserving privacy budget, we introduce an increasing and concave continuation value function V(B), where *B* denotes the remaining privacy budget. This assumption captures the idea that privacy budget is more valuable when it becomes scarce. In this section, the function is kept general to separate the system model from the algorithmic implementation; a concrete log-based instantiation is introduced later when deriving the budget-prioritized trigger.

The opportunity cost of consuming $\Delta \varepsilon_{i}^{t}$ privacy budget is defined as

(8) $$C_{B}\left(B_{i}^{t},\Delta \varepsilon_{i}^{t}\right)=V\left(B_{i}^{t}\right)-V\left(B_{i}^{t}-\Delta \varepsilon_{i}^{t}\right).$$

This term measures the future value lost by spending privacy budget on the candidate regrouping operation.

### 3.4. Utility Model

The immediate utility of node *i* under group *G* at time *t* is modeled as

(9) $$U_{i}^{t}\left(G\right)=L_{i}^{t}\left(G\right)-\beta_{i}\Phi_{i}^{t}\left(G\right)-\nu D_{i}^{t}\left(G\right),$$

where $L_{i}^{t}\left(G\right)$ denotes the learning or service cooperation gain, $\beta_{i}$ is the privacy-sensitivity coefficient of node *i*, $\Phi_{i}^{t}\left(G\right)$ denotes the privacy-cost term associated with group participation, $D_{i}^{t}\left(G\right)$ denotes the group-dependent service-quality penalty term, and ν≥0 is a generic weight for this penalty term. The product $\nu D_{i}^{t}\left(G\right)$ can be instantiated as a weighted composite of normalized delay, SLA-violation, and load-imbalance proxies. Based on the state representation in Equation (3), $L_{i}^{t}\left(G\right)$ may depend on the learning-related state $d_{i}^{t}$, while $D_{i}^{t}\left(G\right)$ may depend on the workload intensity $\lambda_{i}^{t}$ and the service-quality state $q_{i}^{t}$.

The privacy-cost term $\Phi_{i}^{t}\left(G\right)$ and the budget opportunity cost $C_{B}\left(B_{i}^{t},\Delta \varepsilon_{i}^{t}\right)$ capture different effects. The former represents the immediate privacy-related disutility in the current training or service round, whereas the latter measures the future value lost by consuming limited remaining privacy budget. Thus, $\Phi_{i}^{t}\left(G\right)$ affects the current group utility, while $C_{B}$ affects whether a new structural update should be triggered.

In the proposed BP-DR implementation, privacy awareness is instantiated through the remaining privacy budget and its opportunity cost in the trigger mechanism. The coefficient $\beta_{i}$ is retained as a general modeling parameter to characterize heterogeneous privacy sensitivity, whereas the trace-driven experiments instantiate privacy-aware decisions through budget states and opportunity-cost terms.

The migration or reconfiguration cost $M_{i}^{t}$ is not included directly in $U_{i}^{t}\left(G\right)$, because it is incurred only when the system moves from the current group $G_{i}^{t}$ to a candidate group $G_{i}^{t,\mathrm{new}}$. It is therefore evaluated as a transition cost in the regrouping decision. Under this model, the immediate improvement of a candidate regrouping operation follows the definition in Equation (4). Section 4 introduces the trigger comparison that evaluates this improvement against reconfiguration cost and privacy-budget opportunity cost.

### 3.5. Problem Formulation

The dynamic grouping problem is to determine a sequence of grouping structures ${\left\{\Pi^{t}\right\}}_{t=1}^{T+1}$, with $\Pi^{1}$ fixed by initialization, together with triggered single-node local regrouping decisions over *T* time slots, such that the long-term system utility is improved while respecting privacy-budget feasibility and avoiding excessive reconfiguration.

A general formulation is

(10) $$\max_{{\left\{\Pi^{t+1},z_{i}^{t}\right\}}_{t=1}^{T}}\sum_{t=1}^{T}\sum_{i\in N}\left(U_{i}^{t}\left(G_{i}^{t}\right)-\mu M_{i}^{t}z_{i}^{t}\right),$$

subject to the cumulative privacy-budget constraint in Equation (5), the per-operation feasibility condition in Equation (7), the requirement that $\Pi^{t}$ is a valid partition of N for each *t*, and the one-commit-per-slot constraint $\sum_{i\in N}z_{i}^{t}\leq 1$ for all *t*. Here, $z_{i}^{t}\in \left\{0,1\right\}$ is a binary acceptance indicator for the candidate regrouping operation $a_{i}^{t}$, where $z_{i}^{t}=1$ means that $a_{i}^{t}$ is accepted at time *t*, and $z_{i}^{t}=0$ otherwise. With this constraint, at most one candidate regrouping operation can be committed in each time slot, and migration or reconfiguration cost is charged only when a structural update is actually performed.

This formulation captures the long-term objective of maintaining useful collaboration structures under dynamic conditions. Solving it exactly would require future workload evolution, privacy-budget consumption, group transitions, and learning dynamics, which are generally unavailable in practical edge federated services. Therefore, this paper adopts a triggered local regrouping approach, where candidate operations are evaluated using current utility improvement, migration or reconfiguration cost, and the continuation value of remaining privacy budget, and then ranked according to the resulting trigger surplus. The resulting budget-prioritized trigger is presented in the next section.

## 4. Proposed Budget-Prioritized Dynamic Regrouping Method

This section presents the proposed Budget-Prioritized Dynamic Regrouping (BP-DR) method. Rather than recomputing a global grouping at every time slot, BP-DR evaluates a limited set of local candidate operations and accepts one only when it is privacy-budget feasible and its immediate utility improvement compensates for reconfiguration cost, privacy-budget opportunity cost, and a base anti-oscillation margin. This design follows the event-triggered principle of suppressing unnecessary updates under small state fluctuations [32].

BP-DR first filters candidate operations according to privacy-budget feasibility and then evaluates the trigger surplus of each feasible candidate. The candidate with the largest trigger surplus is selected, and regrouping is committed only when its surplus is non-negative. The trigger accounts for the remaining privacy budget as a continuation resource by incorporating the corresponding budget opportunity cost into the decision threshold. The theoretical rationale of this trigger is presented in Section 5.

### 4.1. Method Overview

**Figure 2:**
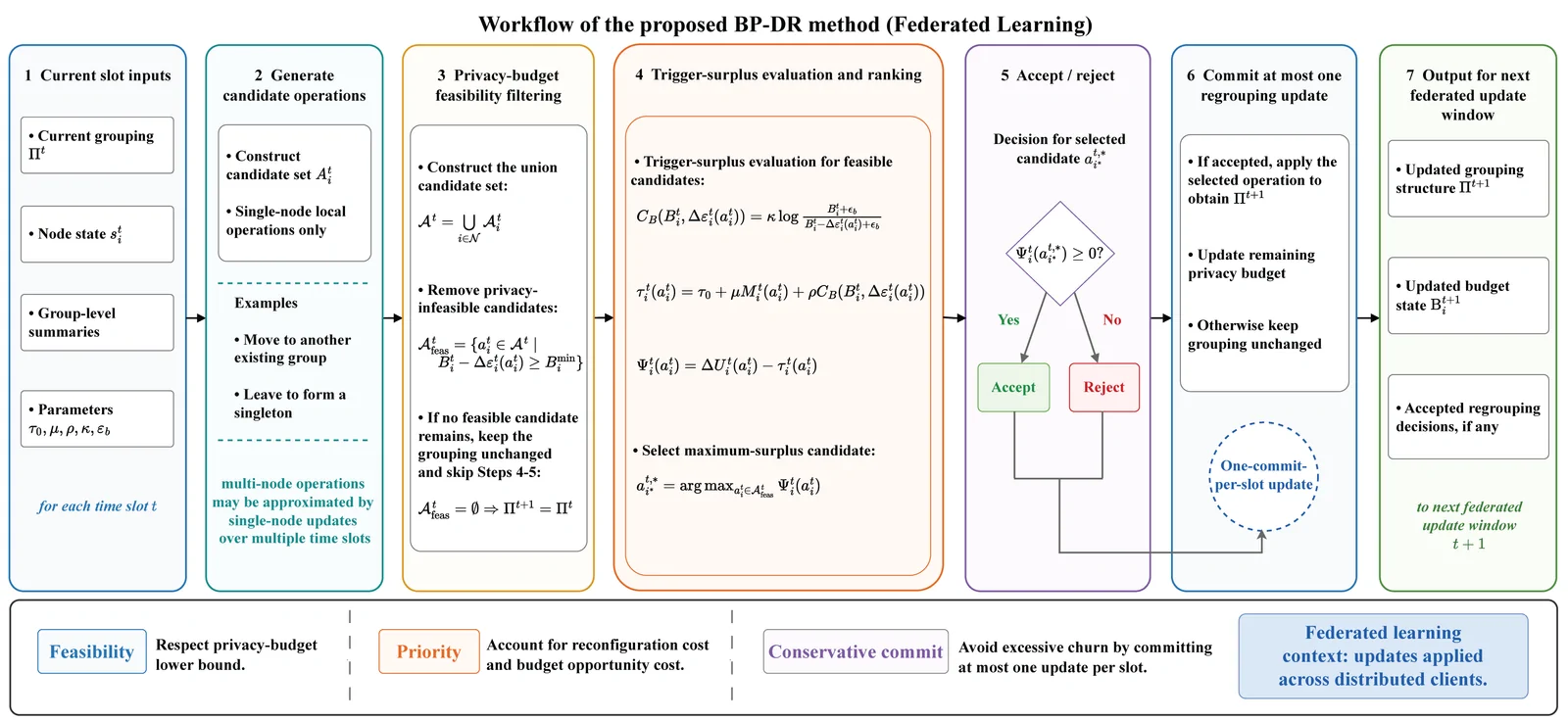
Workflow of the proposed BP-DR method for budget-prioritized dynamic regrouping in federated learning. The superscript * denotes the selected maximum-surplus candidate, while colors are used only to distinguish workflow stages and decision annotations and do not represent additional quantitative information.

BP-DR takes the initial grouping $\Pi^{1}$ as input and performs triggered local regrouping over subsequent time slots. In the experiments, all compared methods use the same initial grouping for fairness. Operating at the regrouping-decision level, BP-DR determines whether the grouping should be updated before the next federated training or service-update window, while the local training, aggregation, and privacy-accounting procedures are specified in the experimental setup. Figure 2 summarizes the workflow from candidate generation and privacy-budget feasibility filtering to trigger-surplus evaluation and candidate ranking, followed by the one-commit-per-slot update.

At each time slot *t*, node *i* observes or estimates its local state $s_{i}^{t}$, including workload intensity, remaining privacy budget, group assignment, service-quality state, and learning-related state. Based on this state and group-level summaries, the system generates a candidate set $A_{i}^{t}$, where each operation $a_{i}^{t}\in A_{i}^{t}$ maps the current group $G_{i}^{t}$ to a candidate group $G_{i}^{t,\mathrm{new}}$. The per-node candidate sets are combined into the union candidate set $A^{t}={⋃}_{i\in N}A_{i}^{t}$. For each candidate, BP-DR first estimates the additional privacy expenditure $\Delta \varepsilon_{i}^{t}\left(a_{i}^{t}\right)$ for privacy-budget feasibility filtering. For each remaining feasible candidate, BP-DR evaluates the immediate utility improvement $\Delta U_{i}^{t}\left(a_{i}^{t}\right)$ and the migration or reconfiguration cost proxy $M_{i}^{t}\left(a_{i}^{t}\right)$ for subsequent trigger-surplus evaluation.

Candidate operations that violate the privacy-budget feasibility condition are removed before ranking. For each remaining candidate, BP-DR evaluates the trigger surplus by accounting for immediate utility improvement, migration or reconfiguration cost, and privacy-budget opportunity cost. The candidate with the largest trigger surplus is selected and accepted only when its surplus is non-negative.

### 4.2. Candidate Local Regrouping Operations

BP-DR considers local regrouping operations rather than recomputing a full global grouping structure. The candidate set is restricted to single-node operations: a node may move to another existing group or leave its current group to form a singleton. This restriction allows utility improvement, privacy expenditure, and reconfiguration cost to be evaluated for the same node. Multi-node operations, such as swaps or split–merge adjustments, are not included in the core algorithm but may be approximated by sequential single-node updates across time slots. Each candidate must result in a valid grouping structure.

For node *i* at time *t*, let $A_{i}^{t}$ denote its candidate operation set. For each $a_{i}^{t}\in A_{i}^{t}$, the resulting candidate group is denoted by $G_{i}^{t,\mathrm{new}}\left(a_{i}^{t}\right)$. The action-dependent utility improvement follows Equation (4), where $G_{i}^{t,\mathrm{new}}$ is instantiated as $G_{i}^{t,\mathrm{new}}\left(a_{i}^{t}\right)$. We denote the resulting quantity by $\Delta U_{i}^{t}\left(a_{i}^{t}\right)$.

To control computational cost, $A_{i}^{t}$ may be restricted to relevant destination groups based on distributional similarity, workload compatibility, or group-level utility estimates. Such a restriction changes only the evaluated candidate set, not the BP-DR trigger rule.

### 4.3. Privacy-Budget Feasibility Filtering

Before trigger evaluation, candidate operations are filtered according to the privacy-budget feasibility constraint. Let $\Delta \varepsilon_{i}^{t}\left(a_{i}^{t}\right)$ denote the estimated additional privacy expenditure required by candidate operation $a_{i}^{t}$. Following Equation (7), its feasibility is evaluated by instantiating $\Delta \varepsilon_{i}^{t}$ as $\Delta \varepsilon_{i}^{t}\left(a_{i}^{t}\right)$.

If the condition is violated, the corresponding candidate operation is removed from further evaluation, preventing the remaining privacy budget from falling below the reserved lower bound. Section 6 specifies the experimental privacy-accounting rule, including the relationship between the estimated expenditure used for feasibility checking and the realized privacy consumption charged after an operation is accepted.

### 4.4. Budget-Prioritized Trigger Evaluation

After privacy-budget feasibility filtering, BP-DR evaluates the trigger surplus of each feasible candidate operation. Following Equation (8), we instantiate $\Delta \varepsilon_{i}^{t}$ as $\Delta \varepsilon_{i}^{t}\left(a_{i}^{t}\right)$ and denote the resulting action-dependent opportunity cost by $C_{B}\;\left(B_{i}^{t},\Delta \varepsilon_{i}^{t}\left(a_{i}^{t}\right)\right)$.

The implemented trigger uses the following log-based continuation-value function:

(11) $$V\left(B\right)=\kappa \log\left(B+\epsilon_{b}\right),\;\kappa >0,\;\epsilon_{b}>0.$$

The corresponding opportunity cost is

(12) $$C_{B}\;\left(B_{i}^{t},\Delta \varepsilon_{i}^{t}\left(a_{i}^{t}\right)\right)=\kappa \log\frac{B_{i}^{t}+\epsilon_{b}}{B_{i}^{t}-\Delta \varepsilon_{i}^{t}\left(a_{i}^{t}\right)+\epsilon_{b}}.$$

The budget-prioritized trigger threshold is defined as

(13) $$\tau_{i}^{t}\left(a_{i}^{t}\right)=\tau_{0}+\mu M_{i}^{t}\left(a_{i}^{t}\right)+\rho C_{B}\;\left(B_{i}^{t},\Delta \varepsilon_{i}^{t}\left(a_{i}^{t}\right)\right),$$

where $\tau_{0}\geq 0$ is the base anti-oscillation margin, μ≥0 weights the reconfiguration cost, and ρ≥0 weights the privacy-budget opportunity cost. Proposition 1 motivates the reconfiguration-cost and privacy-budget opportunity-cost terms through a one-step local comparison, whereas $\tau_{0}$ is an additional algorithmic stability margin and is not part of that derivation. Under the log-based instantiation, ρ and κ enter the threshold as a product; therefore, we fix κ and tune ρ in the experiments.

A privacy-budget-feasible selected candidate is accepted if

(14) $$\Delta U_{i}^{t}\left(a_{i}^{t}\right)\geq \tau_{i}^{t}\left(a_{i}^{t}\right).$$

Equivalently, define the trigger surplus as

(15) $$\Psi_{i}^{t}\left(a_{i}^{t}\right)=\Delta U_{i}^{t}\left(a_{i}^{t}\right)-\tau_{i}^{t}\left(a_{i}^{t}\right).$$

The selected candidate is accepted only when $\Psi_{i}^{t}\left(a_{i}^{t}\right)\geq 0$. In the one-commit-per-slot implementation, BP-DR first filters infeasible candidates, evaluates the trigger surplus of feasible candidates, and then selects the candidate with the maximum surplus for acceptance evaluation. The experimental setup specifies the instantiation of $M_{i}^{t}\left(a_{i}^{t}\right)$, the privacy-accounting rule for $\Delta \varepsilon_{i}^{t}\left(a_{i}^{t}\right)$, and the settings of $\tau_{0},\mu ,\rho ,\kappa ,$ and $\epsilon_{b}$.

### 4.5. Algorithm Procedure

Algorithm 1 summarizes the one-commit-per-slot BP-DR procedure starting from the initial grouping $\Pi^{1}$. Because $\Pi^{1}$ is defined before the first time slot, the updates over *T* time slots produce the grouping sequence $\Pi^{2},\ldots ,\Pi^{T+1}$. Let $i^{*}$ denote the node associated with the selected candidate at time *t*, and let $a_{i^{*}}^{t,*}$ denote the selected operation. Since each candidate operation is associated with a single node, selecting the operation also determines the corresponding node $i^{*}$.

At each time slot, BP-DR generates structurally feasible single-node candidates under $\Pi^{t}$ and removes candidates that violate the privacy-budget feasibility constraint. The trigger surplus of each feasible candidate is then evaluated using Algorithm 2, and the candidate with the largest surplus value is selected. If the selected candidate satisfies the budget-prioritized trigger condition, the grouping is updated once; otherwise, it remains unchanged. Hence, at most one regrouping operation is committed per time slot.

Algorithm 2 is invoked only for candidates that have already passed the privacy-budget feasibility filtering in Algorithm 1. For each feasible candidate, it calculates the utility improvement, estimates the reconfiguration cost and privacy-budget opportunity cost, and derives the trigger threshold and surplus. The resulting surplus value is used for candidate ranking, while the acceptance condition in Algorithm 1 determines whether the selected operation is committed.

**Algorithm 1** BP-DR: One-Commit-per-Slot Budget-Prioritized Dynamic Regrouping
**Input:** Initial grouping $\Pi^{1}$, initial budgets ${\left\{B_{i}^{0}\right\}}_{i\in N}$, reserved budgets ${\left\{B_{i}^{\min}\right\}}_{i\in N}$, parameters $\tau_{0},\mu ,\rho ,\kappa ,\epsilon_{b}$
**Output:** Dynamic grouping sequence ${\left\{\Pi^{t}\right\}}_{t=1}^{T+1}$
  1:Initialize remaining budgets $B_{i}^{1}\leftarrow B_{i}^{0}$ for all i∈N  2:**for** t=1,2,…,T **do**  3:      Observe or estimate node states ${\left\{s_{i}^{t}\right\}}_{i\in N}$  4:      Generate structurally feasible single-node candidate sets ${\left\{A_{i}^{t}\right\}}_{i\in N}$ under the current grouping $\Pi^{t}$  5:      Construct the union candidate set $A^{t}\leftarrow {⋃}_{i\in N}A_{i}^{t}$  6:      Remove candidates violating privacy-budget feasibility constraints:       $A_{\mathrm{feas}}^{t}\leftarrow \left\{a_{i}^{t}\in A^{t}:B_{i}^{t}-\Delta \varepsilon_{i}^{t}\left(a_{i}^{t}\right)\geq B_{i}^{\min}\right\}$  7:      **if** $A_{\mathrm{feas}}^{t}=\emptyset$ **then**  8:            Keep the grouping unchanged: $\Pi^{t+1}\leftarrow \Pi^{t}$  9:      **else**10:            Evaluate the trigger surplus of each candidate in $A_{\mathrm{feas}}^{t}$ using Algorithm 211:            Select the candidate with maximum trigger surplus:     $a_{i^{*}}^{t,*}\leftarrow \arg\max_{a_{i}^{t}\in A_{\mathrm{feas}}^{t}}\Psi_{i}^{t}\left(a_{i}^{t}\right)$12:            **if** $\Psi_{i^{*}}^{t}\left(a_{i^{*}}^{t,*}\right)\geq 0$ **then**13:                 Apply $a_{i^{*}}^{t,*}$ to the current grouping and obtain $\Pi^{t+1}$14:                 Record the corresponding privacy expenditure for budget accounting15:            **else**16:                 Keep the grouping unchanged: $\Pi^{t+1}\leftarrow \Pi^{t}$17:            **end if**18:      **end if**19:      Update remaining privacy budgets according to the realized privacy accounting rule20:
**end for**



**Algorithm 2** Trigger-Surplus Evaluation for a Feasible Candidate
**Input:** Node *i*, time *t*, current grouping $\Pi^{t}$, feasible candidate operation $a_{i}^{t}\in A_{\mathrm{feas}}^{t}$, candidate group $G_{i}^{t,\mathrm{new}}\left(a_{i}^{t}\right)$ induced by $a_{i}^{t}$ under $\Pi^{t}$, state $s_{i}^{t}$, remaining budget $B_{i}^{t}$, estimated privacy expenditure $\Delta \varepsilon_{i}^{t}\left(a_{i}^{t}\right)$, parameters $\tau_{0},\mu ,\rho ,\kappa ,\epsilon_{b}$
**Output:** Trigger surplus $\Psi_{i}^{t}\left(a_{i}^{t}\right)$
1:Compute the utility improvement under $\Pi^{t}$    $\Delta U_{i}^{t}\left(a_{i}^{t}\right)=U_{i}^{t}\;\left(G_{i}^{t,\mathrm{new}}\left(a_{i}^{t}\right)\right)-U_{i}^{t}\left(G_{i}^{t}\right)$2:Estimate the reconfiguration cost $M_{i}^{t}\left(a_{i}^{t}\right)$3:Compute the privacy-budget opportunity cost    $C_{B}\;\left(B_{i}^{t},\Delta \varepsilon_{i}^{t}\left(a_{i}^{t}\right)\right)=\kappa \log\frac{B_{i}^{t}+\epsilon_{b}}{B_{i}^{t}-\Delta \varepsilon_{i}^{t}\left(a_{i}^{t}\right)+\epsilon_{b}}$4:Compute the trigger threshold    $\tau_{i}^{t}\left(a_{i}^{t}\right)=\tau_{0}+\mu M_{i}^{t}\left(a_{i}^{t}\right)+\rho C_{B}\;\left(B_{i}^{t},\Delta \varepsilon_{i}^{t}\left(a_{i}^{t}\right)\right)$5:Compute the trigger surplus    $\Psi_{i}^{t}\left(a_{i}^{t}\right)=\Delta U_{i}^{t}\left(a_{i}^{t}\right)-\tau_{i}^{t}\left(a_{i}^{t}\right)$6:**return** $\Psi_{i}^{t}\left(a_{i}^{t}\right)$


### 4.6. Complexity and Implementation Discussion

Let $R_{i}^{t}=\left|A_{i}^{t}\right|$ denote the number of candidate operations generated for node *i* at time *t*, and let $R=\max_{i,t}R_{i}^{t}$. Candidate evaluation and ranking require computing or estimating the immediate utility improvement and reconfiguration cost for each operation. Privacy-budget feasibility filtering and trigger-surplus evaluation additionally involve constant-time calculations once the required per-candidate estimates are available. If the utility and reconfiguration-cost evaluations take $O(c_{U})$ and $O(c_{M})$ time, respectively, the per-slot ranking complexity is

(16) $$O\;\left(\sum_{i\in N}R_{i}^{t}\left(c_{U}+c_{M}\right)\right).$$

When $R_{i}^{t}\leq R$, this becomes $O(NR\left(c_{U}+c_{M}\right))$.

BP-DR first removes privacy-infeasible candidate operations and then evaluates the trigger surplus of the remaining feasible candidates. The candidate with the maximum trigger surplus is selected for the final acceptance comparison. Once the required estimates are available, the privacy-feasibility check, opportunity-cost computation, threshold evaluation, and surplus comparison each take O(1) time per candidate and therefore do not change the overall asymptotic complexity. Thus, the overall per-slot complexity remains $O(NR\left(c_{U}+c_{M}\right))$. Moreover, since BP-DR commits at most one operation per time slot, no simultaneous-update conflict resolution is required.

This complexity avoids enumerating all possible global partitions and supports repeated regrouping decisions under changing system states. BP-DR nevertheless remains myopic: it relies on current states, estimated utility improvement, reconfiguration cost, and the continuation value of the remaining privacy budget rather than solving the full long-horizon problem. This is consistent with settings where future workload evolution, privacy consumption, and learning dynamics are unavailable.

## 5. Theoretical Analysis

This section provides a lightweight theoretical justification for the budget-prioritized trigger introduced in Section 4. Rather than solving the full long-horizon dynamic grouping problem, we derive the trigger from a one-step local regrouping decision using a continuation-value surrogate for the remaining privacy budget and explain its dependence on reconfiguration cost and budget scarcity.

For notational simplicity, the following analysis considers a fixed feasible candidate operation $a_{i}^{t}$, and the explicit action argument is omitted from action-dependent quantities such as $\Delta U_{i}^{t}\left(a_{i}^{t}\right)$, $M_{i}^{t}\left(a_{i}^{t}\right)$, and $\Delta \varepsilon_{i}^{t}\left(a_{i}^{t}\right)$. The analysis is candidate-wise: BP-DR applies this comparison to each feasible candidate and ranks candidates according to the trigger surplus defined in Equation (15).

### 5.1. Continuation Value and Privacy-Budget Opportunity Cost

The remaining privacy budget is a finite resource that affects not only the current regrouping operation but also future participation in federated training or service updates under the decision-level privacy-resource accounting model. To capture this effect, we use a continuation-value function V(B), where *B* denotes the remaining privacy budget.

**Assumption** **1.** *The continuation-value function V(B) is increasing and concave over the feasible privacy-budget domain.*

The increasing property means that a larger remaining privacy budget provides greater future flexibility. The concavity property captures diminishing marginal returns: losing a unit of budget becomes more costly when the remaining budget is already small. For a candidate regrouping operation that requires an estimated additional privacy expenditure $\Delta \varepsilon_{i}^{t}$, the privacy-budget opportunity cost is given by Equation (8). This term measures the future value lost by spending privacy budget on the candidate regrouping operation.

Before entering the trigger comparison, the candidate operation must also satisfy the privacy-budget feasibility condition in Equation (7). If this condition is violated, the operation is infeasible and is discarded directly.

### 5.2. Two-Option Local Regrouping Decision

At time slot *t*, node *i* evaluates whether to stay in its current group $G_{i}^{t}$ or accept a candidate regrouping operation that leads to $G_{i}^{t,\mathrm{new}}$. The corresponding immediate utility improvement is defined in Equation (4). We consider a one-step decision model with two options.

**Stay option.** If node *i* stays in its current group, its one-step surrogate value is

(17) $$Q_{i}^{t}\left(\mathrm{stay}\right)=U_{i}^{t}\left(G_{i}^{t}\right)+\rho V\left(B_{i}^{t}\right),$$

where ρ≥0 controls the weight assigned to the remaining-budget continuation value and, equivalently, to the resulting privacy-budget opportunity cost in the trigger.

**Regroup option.** If node *i* accepts the candidate regrouping operation, it obtains the utility of the candidate group, pays the reconfiguration cost, and consumes additional privacy budget. Its one-step surrogate value is

(18) $$Q_{i}^{t}\left(\mathrm{regroup}\right)=U_{i}^{t}\left(G_{i}^{t,\mathrm{new}}\right)-\mu M_{i}^{t}+\rho V\left(B_{i}^{t}-\Delta \varepsilon_{i}^{t}\right),$$

where μ≥0 controls the weight of the migration or reconfiguration cost proxy.

The candidate regrouping operation is preferred under this one-step comparison if

(19) $$Q_{i}^{t}\left(\mathrm{regroup}\right)\geq Q_{i}^{t}\left(\mathrm{stay}\right).$$

### 5.3. Threshold-Form Trigger

**Proposition** **1.** *For any feasible candidate regrouping operation satisfying Equation (7), the one-step comparison in Equation (19) is equivalent to the following threshold condition:*

(20) $$\Delta U_{i}^{t}\geq \mu M_{i}^{t}+\rho C_{B}\left(B_{i}^{t},\Delta \varepsilon_{i}^{t}\right).$$

**Proof.**  Substituting Equations (17) and (18) into Equation (19), the regrouping condition becomes

(21) $$U_{i}^{t}\left(G_{i}^{t,\mathrm{new}}\right)-\mu M_{i}^{t}+\rho V\left(B_{i}^{t}-\Delta \varepsilon_{i}^{t}\right)\geq U_{i}^{t}\left(G_{i}^{t}\right)+\rho V\left(B_{i}^{t}\right).$$

Moving terms gives

(22) $$U_{i}^{t}\left(G_{i}^{t,\mathrm{new}}\right)-U_{i}^{t}\left(G_{i}^{t}\right)\geq \mu M_{i}^{t}+\rho \left[V\left(B_{i}^{t}\right)-V\left(B_{i}^{t}-\Delta \varepsilon_{i}^{t}\right)\right].$$

Using the definition of $\Delta U_{i}^{t}$ in Equation (4) and the budget opportunity cost in Equation (8), we obtain Equation (20). Since each step above preserves equivalence, the threshold condition is both necessary and sufficient for the one-step regrouping comparison.    □

**Remark** **1.** *Proposition 1 derives the migration-cost term and the privacy-budget opportunity-cost term from a one-step local regrouping decision. The base margin $\tau_{0}$ used in the implemented trigger is not derived from the two-option value comparison. It is introduced as an explicit anti-oscillation regularization term to suppress frequent regrouping caused by small utility fluctuations, noisy workload estimates, or numerical perturbations. Therefore, the implemented trigger used by the proposed method is*

(23) $$\Delta U_{i}^{t}\geq \tau_{i}^{t},$$

*where*

(24) $$\tau_{i}^{t}=\tau_{0}+\mu M_{i}^{t}+\rho C_{B}\left(B_{i}^{t},\Delta \varepsilon_{i}^{t}\right),\;\tau_{0}\geq 0.$$

*This distinction is important: the two-option comparison motivates a trigger criterion balancing reconfiguration cost and privacy-budget opportunity cost. The coefficients μ and ρ are trade-off weights in the one-step surrogate and can be varied for ablation, whereas $\tau_{0}$ is an algorithmic stability margin.*

### 5.4. Threshold Monotonicity

The threshold in Equation (24) has a natural monotonicity property. It becomes harder to trigger regrouping when reconfiguration is expensive or when the remaining privacy budget is scarce.

**Proposition** **2.** *Assume μ≥0, ρ≥0, and V(B) is increasing and concave. For any fixed feasible $\Delta \varepsilon_{i}^{t}>0$, the trigger threshold $\tau_{i}^{t}$ is nondecreasing in the reconfiguration cost $M_{i}^{t}$ and nonincreasing in the remaining privacy budget $B_{i}^{t}$. Equivalently, the threshold is nondecreasing as the remaining privacy budget becomes tighter.*

**Proof.**  For fixed $\Delta \varepsilon_{i}^{t}$, we view $\tau_{i}^{t}$ as a function of $M_{i}^{t}$ and $B_{i}^{t}$. From Equation (24), for any two reconfiguration costs $M_{1}\leq M_{2}$ under the same remaining budget, we have

(25) $$\tau_{i}^{t}\left(M_{2},B_{i}^{t}\right)-\tau_{i}^{t}\left(M_{1},B_{i}^{t}\right)=\mu \left(M_{2}-M_{1}\right)\geq 0.$$

Thus, a higher reconfiguration cost increases the required utility improvement for accepting a regrouping operation.

Next, consider the budget opportunity cost as a function of a generic remaining budget *B* and a fixed feasible expenditure Δε:

(26) $$C_{B}\left(B,\Delta \varepsilon \right)=V\left(B\right)-V\left(B-\Delta \varepsilon \right).$$

For a concave function, the fixed-length increment V(B)−V(B−Δε) is nonincreasing as the interval [B−Δε,B] shifts to the right, which follows from the nonincreasing chord slopes of concave functions. Therefore, for any two feasible remaining budgets $B_{1}\leq B_{2}$,

(27) $$C_{B}\left(B_{1},\Delta \varepsilon \right)\geq C_{B}\left(B_{2},\Delta \varepsilon \right).$$

Using Equation (24), this implies

(28) $$\tau_{i}^{t}\left(M_{i}^{t},B_{1}\right)-\tau_{i}^{t}\left(M_{i}^{t},B_{2}\right)=\rho \left[C_{B}\left(B_{1},\Delta \varepsilon \right)-C_{B}\left(B_{2},\Delta \varepsilon \right)\right]\geq 0,$$

because ρ≥0. Hence, the trigger threshold is nonincreasing in the remaining budget $B_{i}^{t}$. Equivalently, when the remaining privacy budget becomes tighter, the threshold becomes higher. This proves the claimed monotonicity.    □

This monotonicity supports the budget-prioritized nature of the trigger: when privacy budget is abundant, regrouping is mainly limited by utility improvement and reconfiguration cost; when privacy budget becomes scarce, consuming additional budget requires a larger immediate utility gain.

### 5.5. Special Cases and Log-Based Instantiation

The weighted trigger in Equation (24) also provides a clean way to recover several baseline or ablation variants.

**Corollary** **1.** *Different parameter settings of $\tau_{i}^{t}$ correspond to interpretable trigger variants:*

- *$\mu >0,\rho >0,\tau_{0}>0$: the full budget-prioritized trigger, considering reconfiguration cost, privacy-budget opportunity cost, and anti-oscillation margin.*
- *ρ=0: a migration-aware trigger without the privacy-budget opportunity term.*
- *μ=0: a budget-aware trigger without the reconfiguration-cost term.*
- *$\mu =0,\rho =0,\tau_{0}>0$: a fixed-margin trigger.*
- *$\mu =0,\rho =0,\tau_{0}=0$: a utility-improvement-only variant, for which the trigger surplus reduces to $\Psi_{i}^{t}=\Delta U_{i}^{t}$.*

These special cases will be used in the ablation study to isolate the effects of the budget opportunity term, the reconfiguration-cost term, and the base anti-oscillation margin. Under Algorithm 1, this variant ranks feasible candidates by immediate utility improvement and commits the maximum-improvement candidate only when its improvement is non-negative.

Substituting the log-based continuation-value function in Equation (11) into the general opportunity-cost definition in Equation (8) yields the closed-form expression in Equation (12). The adopted continuation-value function is increasing and concave, and the resulting opportunity cost penalizes budget consumption more strongly when the remaining privacy budget is small. The following corollary records the monotonicity of this log-based implementation. Substituting the closed-form opportunity-cost expression in Equation (12) into the threshold definition in Equation (24) yields

(29) $$\tau_{i}^{t}=\tau_{0}+\mu M_{i}^{t}+\rho \kappa \log\frac{B_{i}^{t}+\epsilon_{b}}{B_{i}^{t}-\Delta \varepsilon_{i}^{t}+\epsilon_{b}}.$$

**Corollary** **2.** *For the log-based instantiation adopted in Equation (11), the budget opportunity cost decreases with $B_{i}^{t}$ for any fixed feasible $\Delta \varepsilon_{i}^{t}>0$. Therefore, the corresponding trigger threshold increases as the remaining privacy budget becomes tighter.*

**Proof.**  For simplicity, denote $B=B_{i}^{t}$ and $\Delta \varepsilon =\Delta \varepsilon_{i}^{t}$. Rewriting Equation (12) under this notation gives

(30) $$C_{B}\left(B,\Delta \varepsilon \right)=\kappa \log\frac{B+\epsilon_{b}}{B-\Delta \varepsilon +\epsilon_{b}}.$$

Under the feasibility condition and $\epsilon_{b}>0$, both $B+\epsilon_{b}$ and $B-\Delta \varepsilon +\epsilon_{b}$ are positive. Taking the derivative with respect to *B*, we have

(31) $$\frac{\partial C_{B}\left(B,\Delta \varepsilon \right)}{\partial B}=\kappa \left(\frac{1}{B+\epsilon_{b}}-\frac{1}{B-\Delta \varepsilon +\epsilon_{b}}\right)=-\frac{\kappa \Delta \varepsilon }{\left(B+\epsilon_{b}\right)\left(B-\Delta \varepsilon +\epsilon_{b}\right)}<0.$$

Thus, for a fixed feasible Δε, the opportunity cost decreases as *B* increases and increases when the remaining budget becomes scarce. Since ρ≥0, the threshold increases under budget scarcity.    □

The analysis provides a one-step, continuation-value-based justification rather than a proof of global dynamic optimality. Solving the full dynamic problem would require transition models for workload evolution, privacy-budget consumption, group-structure changes, and learning dynamics, which are beyond the scope of this paper. BP-DR instead provides a lightweight and interpretable trigger that commits the selected candidate only when its immediate utility improvement compensates for reconfiguration cost, privacy-budget opportunity cost, and the base anti-oscillation margin.

## 6. Experimental Evaluation

This section investigates the proposed BP-DR method through trace-driven dynamic simulations and an end-to-end federated training validation. The experiments are designed to address the following questions: (i) whether budget-prioritized dynamic regrouping affects long-term utility under workload evolution; (ii) whether BP-DR can reduce unnecessary regrouping and migration overhead compared with periodic or migration-aware baselines; (iii) how the privacy-budget opportunity term affects budget-related behavior and utility efficiency under different budget conditions; and (iv) how the observed performance varies under workload drift, migration and churn stress, ablation variants, privacy-budget scarcity, and different logical-client scales. Additional analyses examine candidate ranking behavior and workload dynamics, while the FLamby-based validation assesses the integration of BP-DR with practical group-aware federated training.

### 6.1. Experimental Setup

#### 6.1.1. Workloads, Dynamic Scenarios, and Experimental Environment

We adopt Alibaba Cluster Trace 2018 [36] as the base workload trace. The batch-task records are aggregated into arrival-intensity and workload-pressure sequences, from which heterogeneous logical edge nodes are derived. Each logical client is treated as a participating edge node in the system model. The resulting workload sequence consists of 2553 time slots or update windows. Unless otherwise stated, the default setting uses N=8 logical edge nodes and K=4 initial groups, while the scale-sensitivity study considers (N,K)∈{(4,2),(8,2),(8,4),(12,2),(12,4),(16,4),(32,8),(64,16)}. All trace-driven simulation results are averaged across ten independent evaluation seeds {0,1,…,9}. In addition to reporting mean performance and standard deviations over repeated runs, we compute 95% confidence intervals from seed-level outcomes, which are provided in the Supplementary Materials to further characterize run-to-run variability. Because no formal hypothesis tests are performed, small differences in mean performance relative to the observed run-to-run variability are interpreted descriptively rather than as evidence of statistical superiority.

For reproducibility, the trace-driven simulator reads the aggregated arrival_count and workload_sum sequences. Negative values are clipped to zero, and workload pressure is obtained by dividing workload_sum by its maximum positive value over the full sequence. For each run seed, the aggregate arrival and workload signals are decomposed into *N* logical-node signals using seed-dependent Dirichlet base shares with sinusoidal temporal modulation. The shares are renormalized at every time slot so that the logical-node decomposition preserves the corresponding aggregate trace level.

For the end-to-end FL validation, we adopt FLamby Fed-Heart-Disease [37] as the federated training benchmark. Alibaba Cluster Trace 2018 is not used as the FL training dataset; it supplies only the workload windows for regrouping decisions. The Alibaba-derived sequence is normalized using the maximum positive arrival count and workload sum, and then divided into 50 consecutive, approximately equal-length segments. The segment-level mean arrival pressure and workload pressure form the workload window for each FL round.

The full trace-driven sequence is used as the main dynamic workload. We additionally construct stable, burst, mixed-shift-burst, and oscillating-burst scenarios to evaluate workload-drift robustness and migration or churn stress. For the post hoc entropy analysis in Section 6.7, abrupt-shift and gradual-drift variants are also included to characterize additional temporal workload patterns. Table 3 summarizes the main experimental settings.

The stable scenario introduces no additional perturbation. Abrupt-shift applies a step change to a subset of four logical nodes from the midpoint onward, whereas gradual-drift changes the corresponding node weights progressively from T/4 to 3T/4 before fixing them at their final values. Burst and mixed-shift-burst apply deterministic role-specific perturbations to the logical-node arrival and workload signals. All perturbed drift scenarios are renormalized per slot to preserve the aggregate arrival and workload levels. For migration and churn stress, oscillating-burst applies sinusoidal hotspot modulation with period $\max\left(24,\left\lfloor T/20\right\rfloor \right)$ and amplitudes 0.55 and 0.35.

All experiments were implemented in Python 3.11.5 and conducted on a server equipped with an Intel Xeon Gold 6148 CPU at 2.40 GHz and approximately 251 GiB of RAM. PyTorch 2.12.1 and FLamby 0.0.1 were used for the FLamby-based federated training validation. All runs were executed on CPU without GPU acceleration.

#### 6.1.2. BP-DR Implementation and Parameter Settings

BP-DR operates at the regrouping-decision level. At each time slot, it examines single-node move and leave operations under the current grouping, filters structurally feasible and privacy-budget feasible candidates, ranks the remaining candidates according to the trigger-surplus function, and commits at most one regrouping update following Algorithm 1. The selected candidate is accepted only when the trigger condition in Equation (14) is satisfied.

All methods within the same experiment start from the same deterministic balanced initial grouping. In the trace-driven simulations, logical edge nodes are partitioned by their indices to form equal-size groups for all tested (N,K) settings. The initial grouping is fixed across compared methods and run seeds.

The implemented threshold follows Equation (13), where $\tau_{0}$, μ, and ρ control the anti-oscillation margin, migration or reconfiguration cost, and privacy-budget opportunity cost, respectively. The calibration grid covers $\tau_{0}\in \left\{0.0005,0.001,0.005,0.01,0.05\right\}$, μ∈{0.05,0.1,0.5,1.0}, and ρ∈{0.1,0.5,1.0}. Calibration uses seeds {100,…,109} under (N,K)=(8,4), which are disjoint from the formal evaluation seeds. Candidate configurations are ranked by mean migration-adjusted utility, with lower migration count, higher final budget ratio, and lexicographically smaller $\left(\tau_{0},\mu ,\rho \right)$ used as successive tie-breakers. Calibration is performed on the default trace-driven workload without additional drift or churn scenario perturbations. The trigger parameters used in the reported experiments are fixed at $\tau_{0}=0.0005$, μ=0.05, and ρ=0.1. For the log-based continuation value in Equation (11), we set κ=1.0 and $\epsilon_{b}=10^{-12}$. Because ρ and κ enter the threshold as a product, ρ controls the effective strength of the budget opportunity term.

In the trace-driven implementation, $\alpha_{i}$ is used as a service-capacity share proxy rather than as a privacy-accounting parameter. We use the heterogeneous profile $\alpha_{i}\in \left\{0.85,0.95,1.05,1.15\right\}$. For a group *G*, its normalized capacity share is instantiated as

$$c\left(G\right)=\frac{\sum_{i\in G}\alpha_{i}}{\sum_{j\in N}\alpha_{j}}.$$

The coefficients are assigned using deterministic seed-based shuffling for the tested scale settings and the FLamby validation. Homogeneous-alpha Dynamic instead replaces them with their common mean $\bar{\alpha }=1.0$. This implementation-side $\alpha_{i}$ is distinct from the conceptual privacy-sensitivity coefficient $\beta_{i}$ in Equation (9). The two coefficients represent different types of node heterogeneity and have no one-to-one mapping in the experiments. Specifically, $\beta_{i}$ describes the sensitivity of a node’s utility to privacy-related loss in the general formulation, whereas the trace-driven implementation focuses on budget-aware regrouping decisions through the remaining budget $B_{i}^{t}$, additional expenditure $\Delta \varepsilon_{i}^{t}\left(a\right)$, and the privacy-budget opportunity cost $C_{B}\left(B_{i}^{t},\Delta \varepsilon_{i}^{t}\left(a\right)\right)$. Therefore, the privacy-related effect represented by $\beta_{i}\Phi_{i}^{t}\left(G\right)$ in the general formulation is not separately instantiated in the system-level proxy utility; instead, privacy-aware regrouping is implemented through the budget state and opportunity-cost terms in the trigger mechanism.

For reproducibility, the trace-driven experiments instantiate utility at the system level rather than separately evaluating every conceptual term in Equation (9). For each group *G*, let

$$\ell_{G}^{t}=\sum_{i\in G}w_{i}^{t},\;u_{G}^{t}=\frac{\ell_{G}^{t}}{\max\{c\left(G\right),10^{-12}\}},$$

where $w_{i}^{t}$ denotes the normalized workload pressure of node *i*. The served-load, delay, SLA-violation, and load-imbalance proxies are defined as

$$S^{t}=\sum_{G\in \Pi^{t}}\min\left\{\ell_{G}^{t},c\left(G\right)\right\},$$

$$D_{\mathrm{delay}}^{t}=\frac{1}{K_{t}}\sum_{G\in \Pi^{t}}{\left[u_{G}^{t}-1\right]}_{+},\;D_{\mathrm{SLA}}^{t}=\frac{1}{K_{t}}\sum_{G\in \Pi^{t}}{\left[\ell_{G}^{t}-c\left(G\right)\right]}_{+},$$

and

$$D_{\mathrm{bal}}^{t}={\mathrm{Var}}_{G\in \Pi^{t}}\left(u_{G}^{t}\right),$$

where ${\left[x\right]}_{+}=\max\left\{x,0\right\}$. The implemented base utility is

$$U_{\mathrm{base}}^{t}=S^{t}-0.5D_{\mathrm{delay}}^{t}-0.5D_{\mathrm{SLA}}^{t}-0.2D_{\mathrm{bal}}^{t}.$$

For a candidate operation *a*, the utility improvement used by the regrouping decision is

$$\Delta U^{t}\left(a\right)=U_{\mathrm{base}}^{t}\left(\Pi^{t}⊕a\right)-U_{\mathrm{base}}^{t}\left(\Pi^{t}\right).$$

Accepted regrouping operations update the corresponding budget state according to the common privacy-accounting rule. The additional expenditure $\Delta \varepsilon_{i}^{t}\left(a_{i}^{t}\right)$ conservatively estimates the privacy expenditure associated with accepting the candidate operation and entering the subsequent update window, and the same budget-accounting and migration-cost definitions are used across methods whenever applicable. In the current implementation, the privacy-budget quantities are used for decision-level accounting at the regrouping layer; establishing an end-to-end differential-privacy guarantee would require integration with a formal privacy accountant. We set $B_{i}^{\min}=0$. In the migration and churn stress experiment, the migration-cost proxy is defined as $M_{i}^{t}\left(a\right)=s_{M}{\bar{M}}_{i}^{t}\left(a\right)$, where $s_{M}$ is the experimental migration-cost scale and ${\bar{M}}_{i}^{t}\left(a\right)$ is the number of logical edge nodes whose group assignment changes under candidate operation *a*.

For the FLamby validation, we use N=20 logical clients and K=4 initial groups. The center-level samples are split into 20 logical clients using partition seed 2026. With initial-group seed 2027, the clients are deterministically shuffled and divided into four groups of five; workload-mapping seed 2028 is used to construct their round-level workload states. Each current group maintains a group-level federated model. In each round, clients train from their current group model, and their updates are aggregated within the group using training-sample-size weights. The regrouping decision is made after local training and pooled evaluation, and any accepted operation takes effect in the next round. Local training uses Adam with a learning rate of $10^{-3}$, batch size 4, and one local epoch. The FLamby validation results are averaged over ten independent seeds.

#### 6.1.3. Compared Methods

Because existing dynamic clustered FL, adaptive privacy-budget allocation, and migration-aware FL methods rely on different assumptions, we adapt them to a unified BP-DR simulation setting. All methods use the same initial grouping, workload sequence, privacy-budget accounting framework, and evaluation metrics whenever applicable, while adapted baselines retain their method-specific privacy-spending rules.

The compared methods are as follows.

- **Static grouping**. The initial grouping remains unchanged throughout the time horizon, providing a non-regrouping baseline.
- **Periodic regrouping**. Regrouping is performed at fixed intervals, representing a simple time-driven reconfiguration heuristic.
- **Homogeneous-alpha Dynamic**. Dynamic regrouping is applied after replacing the heterogeneous service-capacity coefficients $\left\{\alpha_{i}\right\}$ with their common mean $\bar{\alpha }=1.0$, thereby isolating the effect of service-capacity heterogeneity.
- **Hermes–FedCM-style adapted**. This baseline is inspired by switching-cost-aware and migration-aware FL studies [25,26]. It accounts for migration or reconfiguration cost but does not treat the remaining privacy budget as a continuation resource.
- **Adaptive-DP-spending adapted**. This privacy-budget-aware baseline is inspired by adaptive budget-allocation and DP-aware FL studies [18,19,23], but does not jointly account for budget opportunity cost and migration cost in the trigger.
- **BP-DR**. The proposed method filters privacy-budget feasible candidates, ranks them according to the trigger-surplus function, and accepts the best candidate only when the corresponding trigger condition is satisfied.

These adapted baselines are not exact reproductions of Hermes, FedCM, or existing adaptive DP-spending systems. They are implemented in the same dynamic regrouping setting so that the comparison focuses on their core decision logic.

For implementation consistency, all dynamic methods use the same structurally feasible single-node move and leave candidate set, moved-client privacy-budget feasibility check, migration-cost proxy, and one-commit-per-slot rule. Periodic regrouping evaluates candidates at fixed intervals of 12 time slots and commits the best candidate only when its utility gain net of the unweighted migration cost is positive. Homogeneous-alpha Dynamic otherwise follows the BP-DR decision rule while replacing the heterogeneous service-capacity coefficients with their common mean. Hermes–FedCM-style adapted ranks and accepts candidates using the migration-only score $\Delta U_{i}^{t}\left(a_{i}^{t}\right)-\mu M_{i}^{t}\left(a_{i}^{t}\right)$, which corresponds to the migration-cost-only case of Equation (20), while omitting the base anti-oscillation margin and the privacy-budget opportunity term. Adaptive-DP-spending adapted adjusts background privacy spending according to workload pressure and the remaining-budget ratio, and uses migration-aware candidate scoring without the privacy-budget opportunity term. BP-DR ranks the budget-feasible candidates using the trigger-surplus score Ψ(a), which incorporates utility improvement, migration cost, and privacy-budget opportunity cost.

The FLamby validation compares Static grouping, Hermes–FedCM-style adapted, Adaptive-DP-spending adapted, and BP-DR. The No budget term variant is excluded because the contribution of the privacy-budget opportunity term is evaluated separately in the budget-scarcity stress test.

#### 6.1.4. Evaluation Metrics

The primary system metric is migration-adjusted utility, defined as final cumulative utility after accounting for migration or reconfiguration overhead. We also report cumulative utility, migration count, accepted regrouping count, final budget ratio, total budget consumption, and utility per privacy cost to evaluate utility, stability, and privacy-budget behavior. For the privacy-budget scarcity stress test, final budget ratio and utility per privacy cost are emphasized because they directly reflect budget-aware behavior under limited privacy budgets.

For reproducibility, let

$$C_{\mathrm{budget}}=\sum_{i\in N}\left(B_{i}^{0}-B_{i}^{T+1}\right)$$

denote the total system-wide privacy-budget consumption, including both background decision-level expenditure and additional expenditure caused by accepted regrouping operations. The final budget ratio is defined as

$$R_{\mathrm{budget}}=\frac{\frac{1}{N}\sum_{i\in N}B_{i}^{T+1}}{\frac{1}{N}\sum_{i\in N}B_{i}^{0}}.$$

Since all nodes in the reported experiments use the same initial budget $B^{0}$, this reduces to

$$R_{\mathrm{budget}}=1-\frac{C_{\mathrm{budget}}}{NB^{0}}.$$

Utility per privacy cost is computed as

$$\eta_{\mathrm{privacy}}=\frac{U_{\mathrm{adj}}}{\max\{C_{\mathrm{budget}},10^{-12}\}},$$

where $U_{\mathrm{adj}}$ denotes the migration-adjusted cumulative utility.

Workload-related proxies, including load imbalance, delay, and SLA violation, are recorded as diagnostic signals when applicable. Decision overhead is reported in the scale-sensitivity and FLamby validation experiments. It includes candidate generation and ranking, trigger evaluation, budget-feasibility checking, and commit-decision time, but excludes local model training and pooled evaluation.

For the FLamby validation, pooled test accuracy and loss are additionally reported as mean ± standard deviation over ten independent evaluation seeds. These learning metrics are separated from the system utility metrics, while migration-adjusted utility follows the same definition as in the trace-driven experiments.

### 6.2. Main Comparison

Table 4 presents the overall comparison under the default trace-driven setting. Static grouping obtains the lowest mean cumulative utility and migration-adjusted utility because it cannot adapt the grouping structure to workload evolution. Periodic regrouping provides only limited improvement due to its fixed reconfiguration schedule. In contrast, the dynamic methods generally achieve higher mean utility by adapting group structures to workload variations.

Homogeneous-alpha Dynamic achieves a mean cumulative utility of 317.767 and a migration-adjusted utility of 317.738 under the homogeneous service-capacity profile. Adaptive-DP-spending adapted obtains a lower mean migration-adjusted utility of 317.324 and a utility per privacy cost of 20.272 under the unified adapted implementation, indicating a different utility–migration–budget behavior from the other dynamic methods in this setting. Hermes–FedCM-style adapted achieves the largest mean migration-adjusted utility of 317.797 among the compared methods.

BP-DR obtains a mean migration-adjusted utility of 317.770, which is close to that of Hermes–FedCM-style adapted relative to the observed run-to-run variability. BP-DR also reduces the average migration count to 2.300 compared with 2.800 for Hermes–FedCM-style adapted. Its mean utility per privacy cost is 22.170, compared with 22.163 for Hermes–FedCM-style adapted, while both methods have the same final budget ratio at the displayed precision. These results are interpreted descriptively rather than as evidence of statistical superiority. Overall, BP-DR maintains a competitive utility level while requiring modestly fewer migration operations in the default setting, reflecting its tradeoff-oriented design rather than consistent maximization of any single metric.

### 6.3. Workload Drift Robustness

To assess workload-drift robustness, Table 5 reports the comparison among methods under stable, burst, and mixed-shift-burst scenarios. These scenarios represent progressively dynamic workload conditions, ranging from stable operation to transient bursts and combined workload shifts. The dynamic methods generally obtain higher mean migration-adjusted utility than Static grouping and Periodic regrouping in the tested scenarios, consistent with the increased adaptability provided by dynamic regrouping under evolving workload conditions.

Homogeneous-alpha Dynamic records higher mean migration-adjusted utility than the non-adaptive baselines, while Adaptive-DP-spending adapted obtains lower utility per privacy cost under the present adapted implementation. This indicates that privacy-spending adaptation alone exhibits a different utility–migration–budget behavior from the other dynamic methods in the tested scenarios. Hermes–FedCM-style adapted achieves competitive utility while explicitly incorporating migration cost into candidate selection.

**Figure 3:**
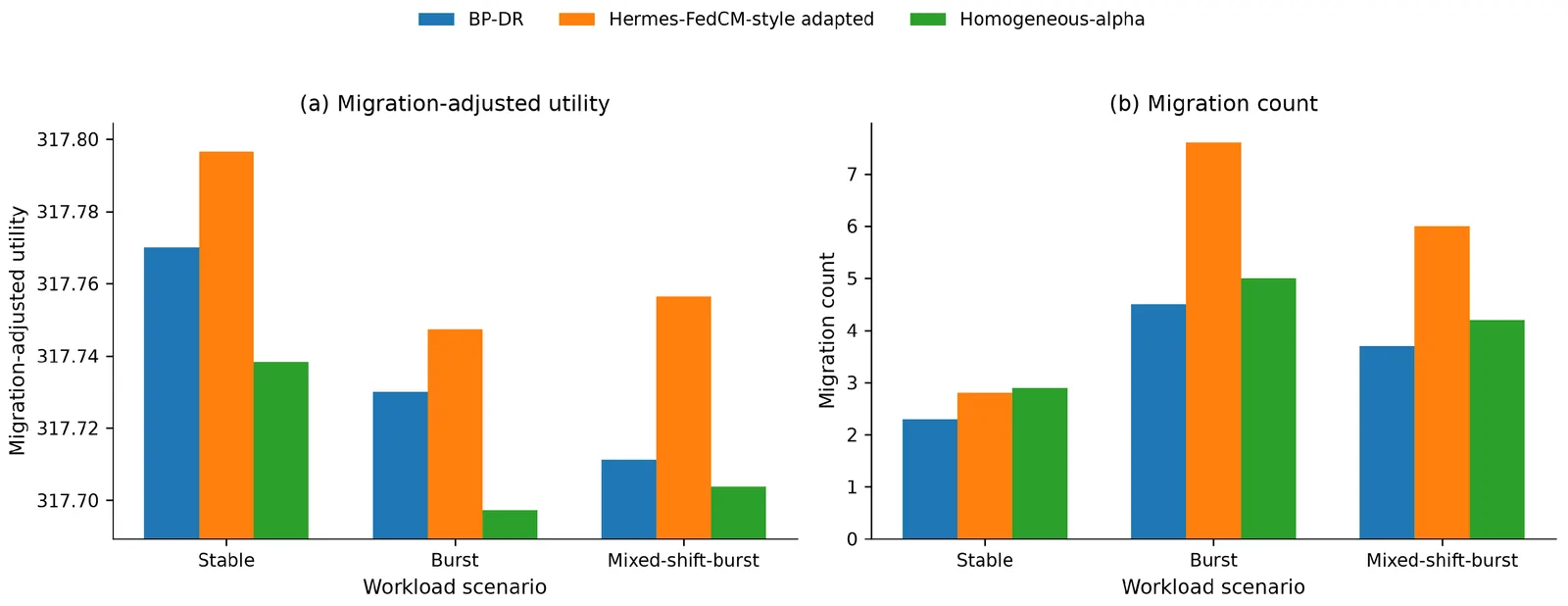
Workload-drift robustness under stable, burst, and mixed-shift-burst scenarios. Panel (**a**) reports the migration-adjusted utility, and Panel (**b**) reports the migration count. BP-DR maintains competitive migration-adjusted utility while reducing migration overhead compared with the Hermes–FedCM-style adapted baseline.

Compared with Hermes–FedCM-style adapted, BP-DR reduces the average migration count from 2.800 to 2.300 in the stable scenario, from 7.600 to 4.500 in the burst scenario, and from 6.000 to 3.700 in the mixed-shift-burst scenario. As shown in Figure 3, BP-DR maintains similar mean utility performance while requiring fewer migration operations under workload variations. These observations suggest that BP-DR favors utility-preserving regrouping decisions rather than reacting to every workload fluctuation, thereby limiting migration activity under the tested workload-drift scenarios.

### 6.4. Migration and Churn Stress

The migration and churn stress experiment varies the migration-cost scale $s_{M}$ under the oscillating-burst scenario, with the results summarized in Table 6. This setting examines whether the methods can maintain a stable utility–migration tradeoff under repeated workload fluctuations and increasing reconfiguration costs. Since the final budget ratios remain close across methods, the analysis mainly focuses on migration-adjusted utility and migration behavior.

Under low and moderate migration costs, BP-DR maintains competitive migration-adjusted utility while reducing migration operations compared with the migration-aware Hermes–FedCM-style adapted baseline. For example, at $s_{M}=0.005$, Hermes–FedCM-style adapted performs 16.700 migrations on average, whereas BP-DR reduces this value to 7.100 with only a marginal difference in utility. Similar trends are observed at $s_{M}=0.010$ and $s_{M}=0.020$, where BP-DR preserves similar utility with fewer regrouping operations.

**Figure 4:**
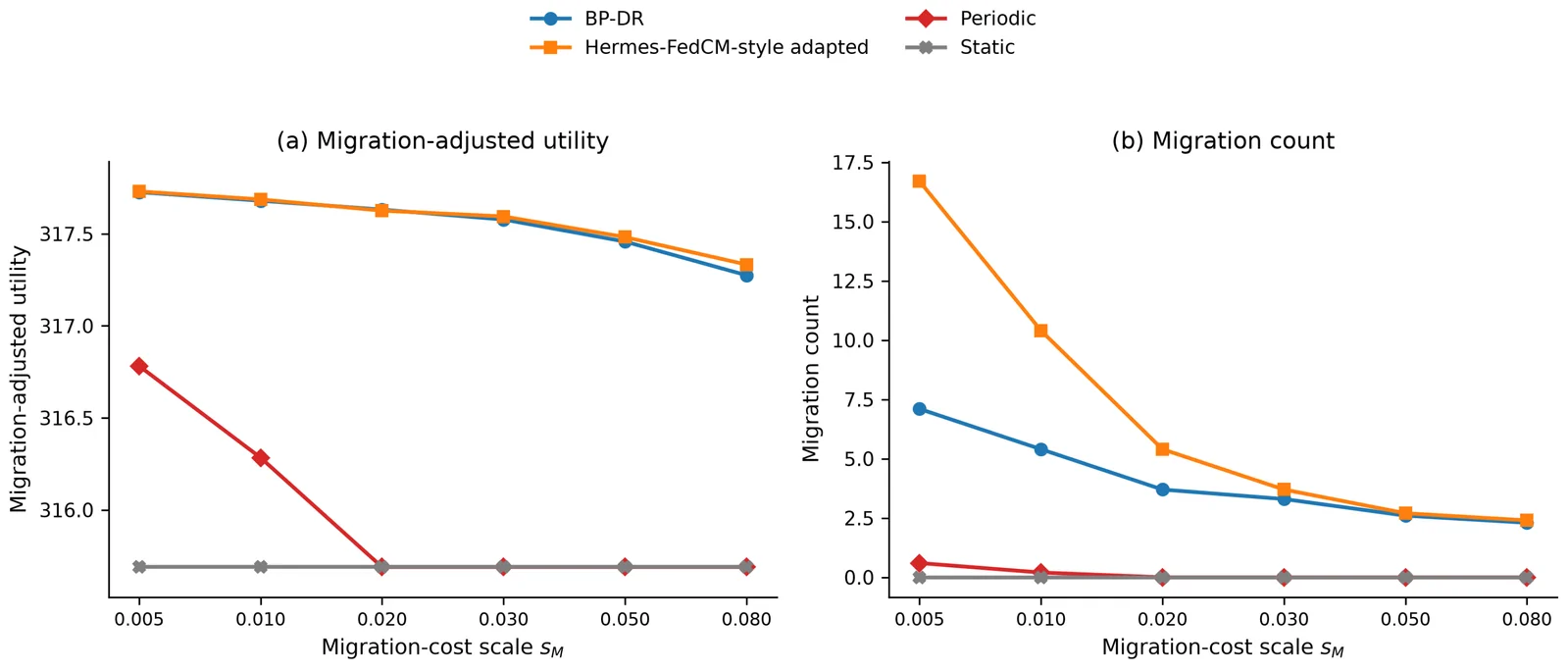
Migration and churn stress under different migration-cost scales. Panel (**a**) shows the migration-adjusted utility, and Panel (**b**) shows the migration count. The results illustrate how BP-DR balances migration-adjusted utility and migration frequency as the migration-cost scale increases.

As the migration-cost scale increases, all dynamic methods become more conservative because frequent reconfiguration becomes less beneficial. BP-DR continues to maintain competitive migration-adjusted utility with controlled migration frequency. In contrast, Static grouping and Periodic regrouping reduce migration by limiting adaptation but obtain lower utility under oscillating workloads. Figure 4 summarizes these utility and migration-count trends across different migration-cost levels.

### 6.5. Ablation and Ranking Analysis

#### 6.5.1. Component Ablation

The component ablation in Table 7 examines the effects of the budget opportunity term, migration-cost term, fixed threshold, and utility-only triggering under three workload scenarios. Unlike the previous baseline comparison, this experiment focuses on the internal design choices of the BP-DR trigger mechanism.

Full BP-DR and the variant without the budget opportunity term produce similar results under the default budget setting because the remaining privacy budgets are sufficient for most decisions. Their performance differences are therefore limited when budget pressure is weak. Removing the migration-cost term or replacing the trigger with a fixed threshold increases migration frequency while retaining comparable cumulative utility, indicating that migration-aware triggering plays an important role in avoiding unnecessary regrouping operations.

**Figure 5:**
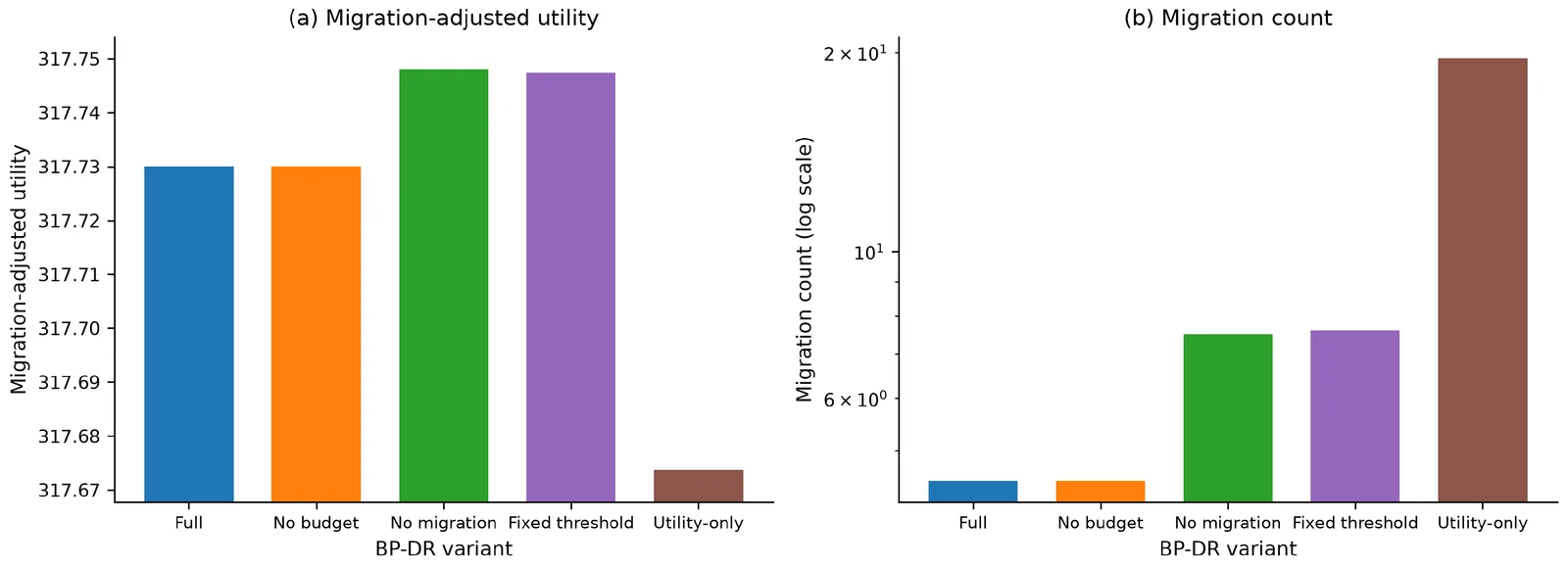
Component ablation results under the burst scenario. Panel (**a**) reports migration-adjusted utility, and Panel (**b**) reports migration count using a logarithmic y-axis. The utility-only trigger causes excessive regrouping, whereas Full BP-DR maintains controlled migration behavior with similar utility.

The Utility-only trigger yields similar cumulative utility but causes substantially more regrouping under the tested scenarios. Its average migration counts increase to 6.2, 19.6, and 18.4 in the stable, burst, and mixed-shift-burst scenarios, respectively, compared with 2.3, 4.5, and 3.7 for Full BP-DR. Under the burst and mixed-shift-burst scenarios, the higher migration frequency results in lower migration-adjusted utility. Figure 5 illustrates this over-regrouping behavior under the burst scenario, where the migration count is presented using a logarithmic scale due to the large difference from other variants.

The variants without the migration-cost term and with a fixed threshold further illustrate the role of the composite trigger design. Under workload perturbations, Full BP-DR requires fewer migration operations while retaining comparable utility. Removing the budget opportunity term has limited impact under the default setting, since its influence becomes more relevant when the available decision-level privacy resource is constrained. Overall, the ablation results distinguish the roles of the migration-aware and budget-aware components, with the migration-cost term exhibiting the clearer effect under the default budget configuration.

#### 6.5.2. Candidate Ranking Behavior Analysis

The candidate ranking analysis in Table 8 compares the proposed trigger-surplus ranking with the utility-first ranking strategy. Unlike the component ablation, this experiment examines whether the proposed ranking changes candidate preferences while preserving the resulting regrouping outcomes.

The two ranking strategies produce identical migration-adjusted utility, migration count, regrouping count, and utility per privacy cost across the tested workload scenarios. Therefore, Table 8 does not demonstrate an aggregate performance advantage for trigger-surplus ranking over utility-first ranking under the evaluated conditions. The non-zero different-selection ratios nevertheless show that the two criteria select different candidates at some intermediate decision points, although these differences do not propagate to the final aggregate metrics.

The role of trigger-surplus ranking should therefore be interpreted as providing a tradeoff-aware candidate-selection mechanism rather than as universally improving aggregate utility. By incorporating migration cost and privacy-budget opportunity cost into the evaluation process, it aligns candidate selection with the BP-DR decision objective even when the final outcomes coincide with those of utility-first ranking in the tested scenarios. The influence of budget awareness under constrained privacy resources is examined separately in the privacy-budget scarcity experiment.

### 6.6. Privacy-Budget Scarcity Evaluation

In the default experiments, the final budget ratios remain high, so the effect of the privacy-budget opportunity term is not fully examined. We therefore investigate budget scarcity under the burst and mixed-shift-burst scenarios by reducing the initial budget from $B_{i}^{0}=1000$ to $B_{i}^{0}\in \left\{2,5,10,20\right\}$ while keeping the other settings unchanged. The results are reported in Table 9.

When the initial budget is relatively sufficient, Full BP-DR and No budget term exhibit similar aggregate behavior because the privacy-budget opportunity cost remains small relative to the other components of the trigger. As the available budget becomes tighter, this term has a greater influence on candidate evaluation by assigning additional value to the remaining decision-level privacy resource.

Under scarce-budget conditions, the aggregate effect of the budget opportunity term is modest and scenario-dependent. For example, under the burst scenario with $B_{i}^{0}=5$, Full BP-DR achieves a migration-adjusted utility of 317.661 with 4.700 migrations, compared with 317.730 and 4.500 migrations for the No budget term. In contrast, under the mixed-shift-burst scenario with $B_{i}^{0}=2$, Full BP-DR performs 3.300 migrations on average, compared with 3.700 for the No budget term, while their final budget ratios and utility-per-privacy values remain close. Thus, the budget-aware term changes some regrouping decisions without uniformly improving migration-adjusted utility, migration frequency, or utility per privacy cost.

Overall, the privacy-budget opportunity term complements hard feasibility filtering by providing an additional opportunity-cost signal for candidate evaluation. Its influence is limited when decision-level privacy resources are abundant and becomes more visible as the available budget is constrained. The results therefore characterize the term as a budget-aware decision component rather than as a mechanism that consistently improves every aggregate performance metric.

### 6.7. Entropy-Based Workload Dynamics Analysis

To further interpret workload dynamics across scenarios, we adopt a normalized entropy indicator for post hoc analysis. Let $\lambda_{i}^{t}$ denote the workload intensity of logical node *i* at time slot *t*, and define its normalized workload share as

$$p_{i}^{t}=\frac{\lambda_{i}^{t}}{\sum_{j=1}^{N}\lambda_{j}^{t}}.$$

The normalized workload entropy is then computed as

$$H_{t}=-\frac{\sum_{i=1}^{N}p_{i}^{t}\ln\left(p_{i}^{t}\right)}{\ln(N)}.$$

Higher values of $H_{t}$ represent a more balanced workload distribution among logical nodes, whereas lower values correspond to stronger workload concentration. This entropy metric is used only for post hoc workload analysis and does not participate in the BP-DR decision process.

**Figure 6:**
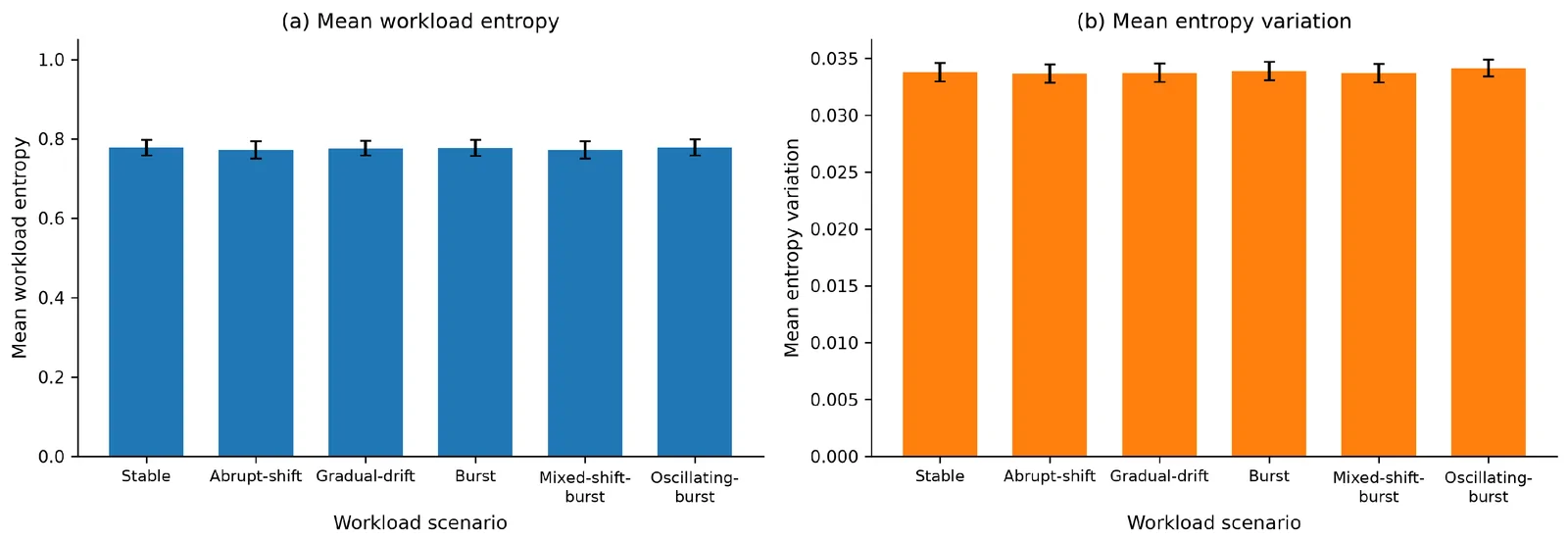
Entropy-based workload dynamics analysis across evaluated scenarios. Panel (**a**) reports the mean workload entropy, and Panel (**b**) reports the mean entropy variation. The results show similar average workload-balance levels across scenarios, with slightly larger temporal variation in the burst and oscillating-burst scenarios.

Figure 6 presents the mean workload entropy and mean entropy variation across the evaluated scenarios. As shown in Figure 6a, the mean workload entropy remains at a similar level across stable, abrupt-shift, gradual-drift, burst, mixed-shift-burst, and oscillating-burst scenarios. This suggests that the overall balance of workload shares does not change substantially across scenarios when averaged over the full time horizon.

The differences become more visible in the entropy-variation results in Figure 6b. Although the variation levels remain close overall, burst and oscillating-burst scenarios exhibit slightly higher mean entropy variation, consistent with more frequent short-term changes in workload distribution. In contrast, the stable and abrupt-shift scenarios show slightly lower variation. These observations are consistent with the intended scenario dynamics.

### 6.8. Scale Sensitivity

Scale sensitivity is assessed by varying the number of logical edge nodes and initial groups, with the results summarized in Table 10. This experiment does not aim to demonstrate large-scale FL training capability; instead, it examines whether the conclusions obtained under the default (N,K)=(8,4) setting remain valid across a range of system scales and grouping configurations. For compactness, the table includes BP-DR together with the strongest non-BP-DR baseline in each configuration.

Under the stable scenario, BP-DR remains close to the strongest baseline across the tested scales. For example, at (N,K)=(12,2), BP-DR obtains a migration-adjusted utility of 317.958, compared with 317.960 for the strongest baseline. As the scale increases to (64,16), the performance gap becomes larger but remains limited relative to the overall utility level, suggesting that the local candidate evaluation strategy maintains competitive decision quality in larger tested configurations.

Under the oscillating-burst scenario, BP-DR remains competitive in migration-adjusted utility across the tested settings. At the default (N,K)=(8,4) configuration, its performance is close to that of the strongest baseline, and a similar competitive pattern is observed in the other tested configurations. These results indicate that BP-DR remains competitive in the tested small-scale and medium-scale dynamic settings, whereas larger decision spaces may allow alternative baselines to obtain slightly higher utility through different regrouping choices.

The decision overhead increases with the scale of the regrouping space because more candidate operations need to be generated and evaluated. It remains below approximately 7 ms for configurations up to (16,4), while larger settings introduce higher overhead due to the increased candidate space. This trend is consistent with the local candidate-ranking complexity discussed in Section 4.6. Overall, BP-DR maintains competitive migration-adjusted utility across the tested scales while highlighting the expected tradeoff between decision quality and computational overhead.

### 6.9. End-to-End Federated Training Validation

Using the 50-round FLamby Fed-Heart-Disease setup described in Section 6.1, we assess whether BP-DR can be integrated with an end-to-end group-aware federated training process. This validation focuses on training compatibility rather than accuracy improvement: FLamby provides the cross-silo federated learning task, while Alibaba Cluster Trace 2018 only provides the workload windows used for regrouping decisions.

Table 11 reports the final learning and system metrics. The mean pooled test accuracy and loss are very similar across the compared methods relative to the observed run-to-run variability. BP-DR records a mean test accuracy of 70.551%, compared with 70.591% for the other methods, while the mean test loss is 0.587 for all methods. These descriptive comparisons do not establish statistical equivalence or a formal non-degradation result. Hermes–FedCM-style adapted has a slightly higher mean migration-adjusted utility, while both Hermes–FedCM-style adapted and BP-DR perform one accepted regrouping operation on average. BP-DR incurs a mean decision overhead of 7.325 ms per decision window. The final budget ratios remain close to 1.000 because the 50-round validation horizon and default budget setting result in limited decision-level privacy expenditure.

**Figure 7:**
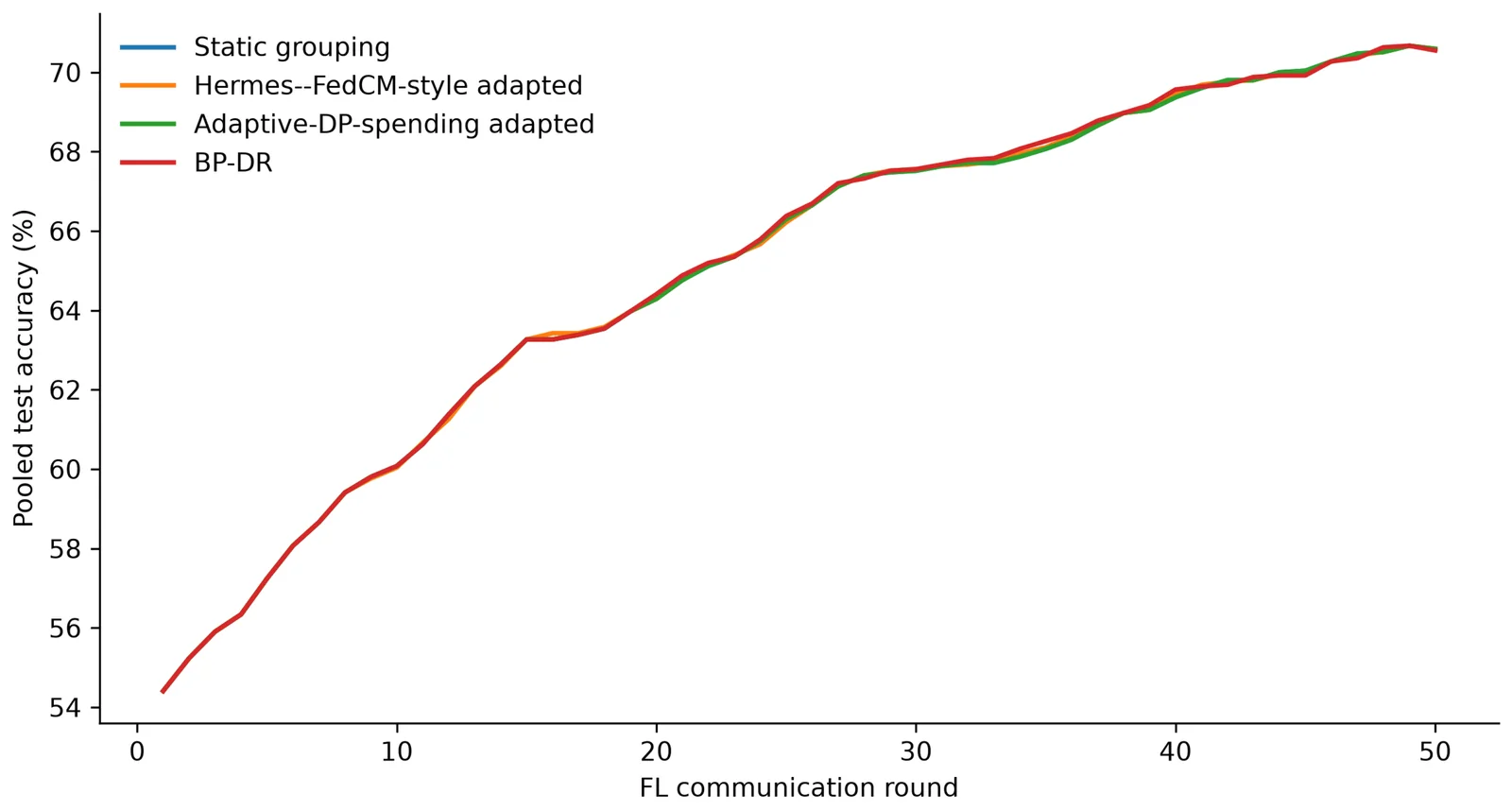
Pooled test accuracy over FL communication rounds in the FLamby Fed-Heart-Disease validation. The compared methods exhibit similar observed convergence trajectories over the tested runs, and this experiment is interpreted primarily as an integration and training-compatibility test.

Figure 7 presents the pooled test accuracy evolution over 50 communication rounds. The compared methods exhibit similar observed convergence trajectories over the tested runs. This experiment is therefore interpreted primarily as an integration and training-compatibility test rather than as evidence of an accuracy benefit from dynamic regrouping or a statistically established absence of degradation. BP-DR retains its regrouping decision capability within the group-aware training workflow, with relatively little regrouping in this validation setting.

### 6.10. Discussion

Taken together, the experiments characterize BP-DR as a tradeoff-oriented method rather than a strategy designed to optimize a single metric. Across the default and workload-drift settings, BP-DR retains competitive mean migration-adjusted utility while often requiring fewer regrouping operations than strong comparison baselines. Because several differences in mean performance are small relative to the observed run-to-run variability, these comparisons are interpreted descriptively rather than as evidence of statistical superiority. The ablation and migration-cost stress experiments further illustrate the role of migration-aware triggering in controlling unnecessary reconfiguration while preserving comparable utility.

The effect of the privacy-budget opportunity term depends on the available decision-level privacy resource. Under budget-abundant conditions, removing this term causes only limited variation because its contribution to the trigger is relatively small. As the available budget becomes constrained, the term has a greater influence on candidate evaluation, although its effects on migration-adjusted utility, migration frequency, and utility per privacy cost remain modest and scenario-dependent. It should therefore be interpreted as an additional opportunity-cost signal rather than as a component that uniformly improves every aggregate metric.

The entropy-based analysis provides a post hoc characterization of workload dynamics and is not part of the BP-DR decision rule. The scale-sensitivity study further indicates that decision overhead increases as the candidate space expands. Accordingly, these results provide evidence that BP-DR remains computationally feasible within the tested simulation configurations, rather than demonstrating real-world large-scale federated-learning scalability.

The FLamby validation is interpreted primarily as an integration and training-compatibility test. The compared methods exhibit similar observed learning metrics, while relatively little regrouping occurs in this setting. Overall, BP-DR provides a local decision mechanism for jointly accounting for utility, reconfiguration cost, and decision-level privacy-resource consumption under the tested dynamic conditions.

## 7. Conclusions

In this paper, we studied budget-prioritized dynamic regrouping for edge federated services under workload drift, privacy-budget constraints, and migration or reconfiguration cost. We proposed BP-DR, a triggered local regrouping method that evaluates single-node candidate operations and commits at most one operation per time slot. By combining privacy-budget feasibility filtering, migration-aware cost control, privacy-budget opportunity cost, and an anti-oscillation margin, BP-DR balances service utility, regrouping stability, and privacy-budget sustainability. The theoretical analysis derives the trigger from a one-step local comparison and shows that it becomes more conservative as migration cost increases or the remaining privacy budget tightens.

Trace-driven experiments based on Alibaba Cluster Trace 2018 indicate that BP-DR maintains competitive mean migration-adjusted utility while often requiring fewer regrouping operations across the tested dynamic and stress settings, although several differences are small relative to the observed run-to-run variability. The privacy-budget opportunity term has limited influence when decision-level privacy resources are abundant and exhibits modest, scenario-dependent effects as the available budget becomes constrained. The FLamby Fed-Heart-Disease validation further provides an integration test in which pooled test accuracy and loss remain similar across the compared methods while BP-DR retains its regrouping capability.

Several limitations remain. The one-commit-per-slot design may be conservative when multiple independent beneficial operations coexist. The theoretical analysis is based on a one-step surrogate comparison and does not provide a formal approximation bound or performance guarantee for the original long-term objective. Privacy expenditure is modeled at the regrouping-decision level and does not constitute a formal end-to-end differential-privacy guarantee, while the FLamby validation covers only one healthcare benchmark. Future work will investigate conflict-free batched regrouping, formal DP-SGD accounting, and larger federated edge deployments with more complex client availability and communication dynamics.

## Figures and Tables

**Table 1:** Comparison with representative works along key dimensions related to dynamic regrouping.

Study	Dyn. Group	Priv. Budget	Mig. Cost	Dynamics	Trigger	Main Criterion
DGDPFL [3]	✓	✓	×	△	△	Budget adjustment
TDCFL [4]	✓	×	×	✓	△	Similarity-driven clustering
FedPLC [5]	✓	×	×	✓	△	Drift-driven adaptation
DecantFed [6]	✓	×	△	△	△	Resource-aware clustering
Kiani et al. [18]	×	✓	×	×	×	Adaptive privacy spending
AWE-DPFL [19]	×	✓	×	×	×	Adaptive budget weighting
Hermes [25]	△	×	✓	△	△	Switching-cost-aware FL
FedCM [26]	✓	×	△	△	△	Migration-aware clustering
Nowzari et al. [32]	×	×	×	×	✓	Event-triggered updates
**This work**	✓	✓	✓	✓	✓	Budget-prioritized trigger

Notes: ✓ indicates that the aspect is explicitly considered; △ indicates that it is partially or indirectly considered; × indicates that it is not a main focus of the study. Dynamics refers to workload drift, concept drift, distribution shift, or other dynamic system conditions. Bold indicates the proposed method.

**Table 2:** Summary of main notations.

Notation	Description
N	Set of participating nodes.
*N*	Number of participating nodes.
*t*, *T*	Time slot index and total number of time slots.
$\Pi^{t}$	Grouping structure at time *t*.
$G_{k}^{t}$, $K_{t}$	The *k*-th group in $\Pi^{t}$ and the number of groups at time *t*.
$G_{i}^{t}$	Current group containing node *i* at time *t*.
$G_{i}^{t,\mathrm{new}}$	Candidate group for node *i* after a regrouping operation.
$a_{i}^{t}$	Single-node candidate local regrouping operation of node *i* at time *t*.
$s_{i}^{t}$	State of node *i* at time *t*.
$\lambda_{i}^{t}$	Workload intensity or task-arrival pressure of node *i*.
$B_{i}^{t}$, $B_{i}^{0}$	Remaining privacy budget and initial privacy budget of node *i*.
$B_{i}^{\min}$	Reserved lower bound on the remaining privacy budget.
$\varepsilon_{i}^{t}$	Privacy budget consumed by node *i* at time *t*.
$\Delta \varepsilon_{i}^{t}$	Estimated additional privacy expenditure required by a candidate regrouping operation.
$q_{i}^{t}$	Service-quality state of node *i*.
$d_{i}^{t}$	Data and learning state of node *i*.
$U_{i}^{t}\left(G\right)$	Immediate utility of node *i* when it belongs to group *G* at time *t*.
$\Delta U_{i}^{t}$	Immediate utility improvement of a candidate regrouping operation.
$L_{i}^{t}\left(G\right)$	Learning or service cooperation gain of node *i* under group *G*.
$\Phi_{i}^{t}\left(G\right)$	Immediate privacy-related disutility of node *i* under group *G*.
$\beta_{i}$	Privacy-sensitivity coefficient of node *i*.
$D_{i}^{t}\left(G\right)$	Service-quality penalty term of node *i* under group *G*.
ν	Generic weight of the service-quality penalty.
$M_{i}^{t}$	Operation-dependent migration or reconfiguration cost proxy.
μ	Weight of the migration or reconfiguration cost.
V(B)	Continuation value function of a generic remaining privacy budget *B*.
$C_{B}\left(B_{i}^{t},\Delta \varepsilon_{i}^{t}\right)$	Privacy-budget opportunity cost induced by consuming $\Delta \varepsilon_{i}^{t}$.
$\tau_{i}^{t}$	Budget-prioritized trigger threshold for node *i* at time *t*.
$\tau_{0}$	Base anti-oscillation margin.
ρ	Weight of the privacy-budget opportunity cost in the trigger threshold.
$z_{i}^{t}$	Binary acceptance indicator for node *i*’s candidate regrouping operation.
$A_{i}^{t}$	Candidate operation set of node *i* at time *t*.
$\Psi_{i}^{t}\left(a_{i}^{t}\right)$	Trigger surplus of candidate operation $a_{i}^{t}$.

**Table 3:** Experimental settings for trace-driven dynamic regrouping and FL validation.

Item	Setting
Base workload trace	Alibaba Cluster Trace 2018
Trace file	batch_task.csv records
Workload signals	arrival intensity and workload pressure
Simulation horizon	2553 time slots/windows
Random seeds	0–9 for trace-driven experiments; ten seeds for evaluation
Main scenarios	stable, burst, and mixed-shift-burst
Migration and churn stress scenario	oscillating-burst
Entropy-analysis scenarios	abrupt-shift and gradual-drift
Default scale	N=8 logical edge nodes and K=4 initial groups
Scale sensitivity	(N,K) configurations from (4,2) to (64,16) (eight settings)
Initial grouping	deterministic balanced static partition, shared by all compared methods
Service-capacity coefficients	heterogeneous $\alpha_{i}\in \left\{0.85,0.95,1.05,1.15\right\}$; homogeneous baseline uses $\bar{\alpha }=1.0$
Utility penalty weights	$w_{\mathrm{delay}}=0.5$, $w_{\mathrm{SLA}}=0.5$, $w_{\mathrm{balance}}=0.2$
Regrouping implementation	structurally feasible single-node move and leave candidates, method-specific candidate ranking, moved-client budget-feasibility filtering, and at most one committed operation per slot
Default trigger parameters	$\tau_{0}=0.0005$, μ=0.05, ρ=0.1
Log-value parameters	κ=1.0 and $\epsilon_{b}=10^{-12}$
Default initial privacy budget	$B_{i}^{0}=1000$ unless otherwise specified
Privacy-budget scarcity evaluation	Burst and mixed-shift-burst scenarios, N=8, K=4, $B_{i}^{0}\in \left\{2,5,10,20\right\}$
Minimum budget reserve	$B_{i}^{min}=0$ in experiments
Privacy-budget handling	per-slot budget accounting and regrouping-spend feasibility filtering
Migration-cost scales	$s_{M}\in \left\{0.005,0.01,0.02,0.03,0.05,0.08\right\}$ for migration and churn stress
End-to-end FL validation	FLamby Fed-Heart-Disease, N=20, K=4, 50 FL rounds, ten seeds
FL validation training	group-level models with Adam, learning rate $10^{-3}$, batch size 4, and one local epoch

Note: $s_{M}$ is an experimental scaling factor for migration-cost stress and is not a new algorithmic weight. The BP-DR migration-cost weight remains μ.

**Table 4:** Trace-driven main results under the default workload setting (N=8,K=4).

Method	Cum.U	Adj.U	Mig.Count	Reg.Count	Bgt.Ratio	U/Privacy
Static grouping	315.639 ± 2.472	315.639 ± 2.472	0.000 ± 0.000	0.000 ± 0.000	0.998 ± 0.000	22.079 ± 0.173
Periodic regrouping	316.094 ± 1.224	316.093 ± 1.227	0.100 ± 0.316	0.100 ± 0.316	0.998 ± 0.000	22.107 ± 0.097
Homogeneous-alpha Dynamic	317.767 ± 0.134	317.738 ± 0.135	2.900 ± 0.994	2.900 ± 0.994	0.998 ± 0.000	22.148 ± 0.037
Adaptive-DP-spending adapted	317.343 ± 0.320	317.324 ± 0.325	1.900 ± 0.738	1.900 ± 0.738	0.998 ± 0.000	20.272 ± 0.046
Hermes–FedCM-style adapted	317.825 ± 0.121	317.797 ± 0.121	2.800 ± 1.135	2.800 ± 1.135	0.998 ± 0.000	22.163 ± 0.029
BP-DR	317.793 ± 0.133	317.770 ± 0.135	2.300 ± 0.823	2.300 ± 0.823	0.998 ± 0.000	22.170 ± 0.027

Note: All values are reported as mean ± standard deviation over ten independent evaluation seeds. Cum. U, Adj. U, Mig. count, Reg. count, Bgt. ratio, and U/privacy denote cumulative utility, migration-adjusted utility, migration count, accepted regrouping count, final budget ratio, and utility per privacy cost, respectively.

**Table 5:** Workload drift robustness results under stable, burst, and mixed-shift-burst scenarios.

Scenario	Method	Adj.U	Mig.Count	Bgt.Ratio	U/Privacy	LoadImb.
stable	Static grouping	315.639 ± 2.472	0.000 ± 0.000	0.998 ± 0.000	22.079 ± 0.173	0.003 ± 0.004
	Periodic regrouping	316.093 ± 1.227	0.100 ± 0.316	0.998 ± 0.000	22.107 ± 0.097	0.003 ± 0.002
	Homogeneous-alpha Dynamic	317.738 ± 0.135	2.900 ± 0.994	0.998 ± 0.000	22.148 ± 0.037	0.000 ± 0.000
	Adaptive-DP-spending adapted	317.324 ± 0.325	1.900 ± 0.738	0.998 ± 0.000	20.272 ± 0.046	0.001 ± 0.001
	Hermes–FedCM-style adapted	317.797 ± 0.121	2.800 ± 1.135	0.998 ± 0.000	22.163 ± 0.029	0.000 ± 0.000
	BP-DR	317.770 ± 0.135	2.300 ± 0.823	0.998 ± 0.000	22.170 ± 0.027	0.000 ± 0.000
burst	Static grouping	315.594 ± 2.458	0.000 ± 0.000	0.998 ± 0.000	22.076 ± 0.172	0.003 ± 0.004
	Periodic regrouping	316.046 ± 1.213	0.100 ± 0.316	0.998 ± 0.000	22.103 ± 0.096	0.003 ± 0.002
	Homogeneous-alpha Dynamic	317.697 ± 0.135	5.000 ± 2.261	0.998 ± 0.000	22.103 ± 0.063	0.000 ± 0.000
	Adaptive-DP-spending adapted	317.340 ± 0.279	2.100 ± 0.738	0.998 ± 0.000	20.270 ± 0.043	0.001 ± 0.000
	Hermes–FedCM-style adapted	317.747 ± 0.105	7.600 ± 2.221	0.998 ± 0.000	22.076 ± 0.068	0.000 ± 0.000
	BP-DR	317.730 ± 0.114	4.500 ± 2.173	0.998 ± 0.000	22.122 ± 0.042	0.000 ± 0.000
mixed-shift-burst	Static grouping	315.342 ± 2.341	0.000 ± 0.000	0.998 ± 0.000	22.058 ± 0.164	0.004 ± 0.004
	Periodic regrouping	316.025 ± 1.132	0.300 ± 0.483	0.998 ± 0.000	22.085 ± 0.100	0.003 ± 0.002
	Homogeneous-alpha Dynamic	317.704 ± 0.092	4.200 ± 1.932	0.998 ± 0.000	22.125 ± 0.040	0.000 ± 0.000
	Adaptive-DP-spending adapted	317.171 ± 0.322	2.000 ± 0.667	0.998 ± 0.000	20.261 ± 0.041	0.001 ± 0.001
	Hermes–FedCM-style adapted	317.756 ± 0.086	6.000 ± 2.867	0.998 ± 0.000	22.104 ± 0.056	0.000 ± 0.000
	BP-DR	317.711 ± 0.093	3.700 ± 0.823	0.998 ± 0.000	22.139 ± 0.031	0.000 ± 0.000

Note: All values are reported as mean ± standard deviation over ten independent evaluation seeds. Adj. U, Mig. count, Bgt. ratio, U/privacy, and Load imb. denote migration-adjusted utility, migration count, final budget ratio, utility per privacy cost, and mean load-imbalance proxy, respectively.

**Table 6:** Migration and churn stress results under the oscillating-burst scenario.

$s_{M}$	Method	Adj.U	Mig.Count	Bgt.Ratio	U/Privacy
0.005	Static grouping	315.690 ± 2.293	0.000 ± 0.000	0.998 ± 0.000	22.083 ± 0.160
	Periodic regrouping	316.782 ± 0.492	0.600 ± 0.966	0.998 ± 0.000	22.138 ± 0.050
	Homogeneous-alpha Dynamic	317.731 ± 0.129	6.200 ± 2.440	0.998 ± 0.000	22.077 ± 0.049
	Hermes–FedCM-style adapted	317.730 ± 0.090	16.700 ± 9.190	0.998 ± 0.000	21.861 ± 0.238
	BP-DR	317.726 ± 0.077	7.100 ± 3.446	0.998 ± 0.000	22.060 ± 0.099
0.010	Static grouping	315.690 ± 2.293	0.000 ± 0.000	0.998 ± 0.000	22.083 ± 0.160
	Periodic regrouping	316.284 ± 1.104	0.200 ± 0.422	0.998 ± 0.000	22.114 ± 0.089
	Homogeneous-alpha Dynamic	317.689 ± 0.123	4.600 ± 1.350	0.998 ± 0.000	22.099 ± 0.034
	Hermes–FedCM-style adapted	317.686 ± 0.093	10.400 ± 5.777	0.998 ± 0.000	21.965 ± 0.189
	BP-DR	317.679 ± 0.090	5.400 ± 3.098	0.998 ± 0.000	22.109 ± 0.065
0.020	Static grouping	315.690 ± 2.293	0.000 ± 0.000	0.998 ± 0.000	22.083 ± 0.160
	Periodic regrouping	315.690 ± 2.293	0.000 ± 0.000	0.998 ± 0.000	22.083 ± 0.160
	Homogeneous-alpha Dynamic	317.600 ± 0.125	4.000 ± 1.491	0.998 ± 0.000	22.103 ± 0.068
	Hermes–FedCM-style adapted	317.625 ± 0.108	5.400 ± 3.098	0.998 ± 0.000	22.105 ± 0.067
	BP-DR	317.631 ± 0.082	3.700 ± 1.636	0.998 ± 0.000	22.114 ± 0.066
0.030	Static grouping	315.690 ± 2.293	0.000 ± 0.000	0.998 ± 0.000	22.083 ± 0.160
	Periodic regrouping	315.690 ± 2.293	0.000 ± 0.000	0.998 ± 0.000	22.083 ± 0.160
	Homogeneous-alpha Dynamic	317.546 ± 0.157	3.400 ± 1.075	0.998 ± 0.000	22.115 ± 0.042
	Hermes–FedCM-style adapted	317.594 ± 0.092	3.700 ± 1.636	0.998 ± 0.000	22.111 ± 0.067
	BP-DR	317.577 ± 0.109	3.300 ± 1.160	0.998 ± 0.000	22.118 ± 0.043
0.050	Static grouping	315.690 ± 2.293	0.000 ± 0.000	0.998 ± 0.000	22.083 ± 0.160
	Periodic regrouping	315.690 ± 2.293	0.000 ± 0.000	0.998 ± 0.000	22.083 ± 0.160
	Homogeneous-alpha Dynamic	317.396 ± 0.182	2.900 ± 1.101	0.998 ± 0.000	22.115 ± 0.020
	Hermes–FedCM-style adapted	317.482 ± 0.139	2.700 ± 1.160	0.998 ± 0.000	22.122 ± 0.033
	BP-DR	317.457 ± 0.146	2.600 ± 1.265	0.998 ± 0.000	22.120 ± 0.034
0.080	Static grouping	315.690 ± 2.293	0.000 ± 0.000	0.998 ± 0.000	22.083 ± 0.160
	Periodic regrouping	315.690 ± 2.293	0.000 ± 0.000	0.998 ± 0.000	22.083 ± 0.160
	Homogeneous-alpha Dynamic	317.231 ± 0.165	2.300 ± 0.949	0.998 ± 0.000	22.110 ± 0.035
	Hermes–FedCM-style adapted	317.333 ± 0.215	2.400 ± 1.174	0.998 ± 0.000	22.105 ± 0.040
	BP-DR	317.275 ± 0.293	2.300 ± 1.160	0.998 ± 0.000	22.111 ± 0.037

Note: All values are reported as mean ± standard deviation over ten independent evaluation seeds. $s_{M}$, Adj. U, Mig. count, Bgt. ratio, and U/privacy denote the migration-cost scale, migration-adjusted utility, migration count, final budget ratio, and utility per privacy cost, respectively.

**Table 7:** Component ablation results of BP-DR trigger variants.

Scenario	Variant	Cum.U	Adj.U	Mig.Count	BudgetCost	U/Privacy
stable	Full BP-DR	317.793 ± 0.133	317.770 ± 0.135	2.300 ± 0.823	14.333 ± 0.015	22.170 ± 0.027
	No budget term	317.793 ± 0.133	317.770 ± 0.135	2.300 ± 0.823	14.333 ± 0.015	22.170 ± 0.027
	No migration term	317.825 ± 0.121	317.797 ± 0.121	2.800 ± 1.135	14.339 ± 0.019	22.163 ± 0.029
	Fixed-threshold trigger	317.825 ± 0.121	317.797 ± 0.121	2.800 ± 1.135	14.339 ± 0.019	22.163 ± 0.029
	Utility-only trigger	317.862 ± 0.122	317.800 ± 0.108	6.200 ± 2.044	14.364 ± 0.023	22.126 ± 0.037
burst	Full BP-DR	317.775 ± 0.118	317.730 ± 0.114	4.500 ± 2.173	14.363 ± 0.028	22.122 ± 0.042
	No budget term	317.775 ± 0.118	317.730 ± 0.114	4.500 ± 2.173	14.363 ± 0.028	22.122 ± 0.042
	No migration term	317.823 ± 0.109	317.748 ± 0.106	7.500 ± 2.415	14.391 ± 0.048	22.080 ± 0.074
	Fixed-threshold trigger	317.823 ± 0.110	317.747 ± 0.105	7.600 ± 2.221	14.394 ± 0.045	22.076 ± 0.068
	Utility-only trigger	317.870 ± 0.124	317.674 ± 0.123	19.600 ± 5.502	14.455 ± 0.060	21.977 ± 0.094
mixed-shift-burst	Full BP-DR	317.748 ± 0.092	317.711 ± 0.093	3.700 ± 0.823	14.351 ± 0.019	22.139 ± 0.031
	No budget term	317.748 ± 0.092	317.711 ± 0.093	3.700 ± 0.823	14.351 ± 0.019	22.139 ± 0.031
	No migration term	317.816 ± 0.070	317.756 ± 0.086	6.000 ± 2.867	14.375 ± 0.034	22.104 ± 0.056
	Fixed-threshold trigger	317.816 ± 0.070	317.756 ± 0.086	6.000 ± 2.867	14.376 ± 0.035	22.104 ± 0.056
	Utility-only trigger	317.885 ± 0.073	317.701 ± 0.079	18.400 ± 6.467	14.432 ± 0.055	22.014 ± 0.086

Note: All values are reported as mean ± standard deviation over ten independent evaluation seeds. Cum. U, Adj. U, Mig. count, Budget cost, and U/privacy denote cumulative utility, migration-adjusted utility, migration count, total budget consumption, and utility per privacy cost, respectively.

**Table 8:** Candidate ranking behavior comparison between BP-DR and utility-first ranking.

Scenario	Method	Adj.U	Mig.Count	Reg.Count	U/Privacy	DifferentSelection Ratio
stable	BP-DR	317.770±0.135	2.300±0.823	2.300±0.823	22.170±0.027	0.207±0.010
stable	BP-DR utility-first ranking	317.770±0.135	2.300±0.823	2.300±0.823	22.170±0.027	0.207±0.010
burst	BP-DR	317.730±0.114	4.500±2.173	4.500±2.173	22.122±0.042	0.206±0.011
burst	BP-DR utility-first ranking	317.730±0.114	4.500±2.173	4.500±2.173	22.122±0.042	0.206±0.011
oscillating-burst	BP-DR	317.679±0.090	5.400±3.098	5.400±3.098	22.109±0.065	0.204±0.012
oscillating-burst	BP-DR utility-first ranking	317.679±0.090	5.400±3.098	5.400±3.098	22.109±0.065	0.204±0.012

Note: All values are reported as mean ± standard deviation over ten independent evaluation seeds. Adj. U, Mig. count, Reg. count, and U/privacy denote migration-adjusted utility, migration count, accepted regrouping count, and utility per privacy cost, respectively. Different selection ratio denotes the pairwise fraction of decision points at which the BP-DR trigger-surplus ranking and utility-first ranking select different candidates; the same pairwise value is therefore reported for both methods within each scenario.

**Table 9:** Privacy-budget scarcity evaluation under different initial privacy budgets.

Scenario	$B_{i}^{0}$	Method	Adj. U	Mig. Count	Bgt. Ratio	U/ Privacy
burst	2	Full BP-DR	317.567 ± 0.163	4.400 ± 2.459	0.251 ± 0.057	26.664 ± 2.155
		No budget term	317.733 ± 0.113	4.300 ± 2.214	0.252 ± 0.057	26.686 ± 2.153
burst	5	Full BP-DR	317.661 ± 0.193	4.700 ± 2.791	0.641 ± 0.001	22.140 ± 0.037
		No budget term	317.730 ± 0.114	4.500 ± 2.173	0.641 ± 0.001	22.122 ± 0.042
burst	10	Full BP-DR	317.703 ± 0.113	4.800 ± 2.573	0.821 ± 0.000	22.139 ± 0.040
		No budget term	317.730 ± 0.114	4.500 ± 2.173	0.820 ± 0.000	22.122 ± 0.042
burst	20	Full BP-DR	317.715 ± 0.126	4.800 ± 2.573	0.910 ± 0.000	22.138 ± 0.038
		No budget term	317.730 ± 0.114	4.500 ± 2.173	0.910 ± 0.000	22.122 ± 0.042
mixed-shift-burst	2	Full BP-DR	317.521 ± 0.137	3.300 ± 1.418	0.250 ± 0.057	26.599 ± 2.120
		No budget term	317.711 ± 0.093	3.700 ± 0.823	0.250 ± 0.058	26.619 ± 2.138
mixed-shift-burst	5	Full BP-DR	317.637 ± 0.131	3.800 ± 1.229	0.642 ± 0.001	22.164 ± 0.074
		No budget term	317.711 ± 0.093	3.700 ± 0.823	0.642 ± 0.002	22.168 ± 0.095
mixed-shift-burst	10	Full BP-DR	317.685 ± 0.096	3.800 ± 0.789	0.821 ± 0.000	22.138 ± 0.024
		No budget term	317.711 ± 0.093	3.700 ± 0.823	0.821 ± 0.000	22.139 ± 0.031
mixed-shift-burst	20	Full BP-DR	317.699 ± 0.100	3.900 ± 0.738	0.910 ± 0.000	22.136 ± 0.029
		No budget term	317.711 ± 0.093	3.700 ± 0.823	0.910 ± 0.000	22.139 ± 0.031

Note: All values are reported as mean ± standard deviation over ten independent evaluation seeds. $B_{i}^{0}$ denotes the initial privacy budget. Adj. U, Mig. count, Bgt. ratio, and U/privacy denote migration-adjusted utility, migration count, final budget ratio, and utility per privacy cost, respectively.

**Table 10:** Scale sensitivity under stable and oscillating-burst scenarios.

Scenario	*N*	*K*	Best Baseline	Best-Baseline Adj. U	BP-DR Adj. U	BP-DR Ovhd. (ms)
stable	4	2	Homogeneous-alpha Dynamic	317.811 ± 0.167	317.782 ± 0.198	0.579 ± 0.055
stable	8	2	Hermes–FedCM-style adapted	317.941 ± 0.026	317.927 ± 0.023	1.296 ± 0.012
stable	8	4	Hermes–FedCM-style adapted	317.797 ± 0.121	317.770 ± 0.135	2.451 ± 0.160
stable	12	2	Hermes–FedCM-style adapted	317.960 ± 0.026	317.958 ± 0.027	1.992 ± 0.024
stable	12	4	Homogeneous-alpha Dynamic	317.857 ± 0.049	317.820 ± 0.080	4.329 ± 0.094
stable	16	4	Hermes–FedCM-style adapted	317.912 ± 0.026	317.870 ± 0.047	6.478 ± 0.142
stable	32	8	Hermes–FedCM-style adapted	317.861 ± 0.039	317.788 ± 0.078	33.907 ± 0.917
stable	64	16	Hermes–FedCM-style adapted	317.697 ± 0.097	317.552 ± 0.183	203.513 ± 9.828
oscillating-burst	4	2	Hermes–FedCM-style adapted	317.708 ± 0.201	317.718 ± 0.153	0.599 ± 0.052
oscillating-burst	8	2	Hermes–FedCM-style adapted	317.911 ± 0.044	317.897 ± 0.044	1.286 ± 0.024
oscillating-burst	8	4	Homogeneous-alpha Dynamic	317.689 ± 0.123	317.679 ± 0.090	2.435 ± 0.128
oscillating-burst	12	2	Hermes–FedCM-style adapted	317.943 ± 0.028	317.923 ± 0.047	1.980 ± 0.016
oscillating-burst	12	4	Hermes–FedCM-style adapted	317.835 ± 0.075	317.793 ± 0.045	4.449 ± 0.204
oscillating-burst	16	4	Hermes–FedCM-style adapted	317.874 ± 0.040	317.840 ± 0.061	6.453 ± 0.056
oscillating-burst	32	8	Hermes–FedCM-style adapted	317.845 ± 0.027	317.784 ± 0.064	34.500 ± 1.995
oscillating-burst	64	16	Hermes–FedCM-style adapted	317.696 ± 0.080	317.538 ± 0.171	196.243 ± 10.267

Note: All values are reported as mean ± standard deviation over ten independent evaluation seeds. “Best baseline” denotes the strongest non-BP-DR baseline in each configuration. Adj. U and Ovhd. denote migration-adjusted utility and BP-DR decision overhead in milliseconds, respectively. Reported values are averaged over repeated runs. Hermes–FedCM and Homogeneous-alpha denote Hermes–FedCM-style adapted and Homogeneous-alpha Dynamic, respectively.

**Table 11:** End-to-end FLamby Fed-Heart-Disease validation under Alibaba-trace-driven workload dynamics.

Method	Test Acc. (%)	Test Loss	Adj. U	Reg. Count	Bgt. Ratio	Ovhd. (ms)
Static grouping	70.591±2.565	0.587±0.014	6.199±0.000	0.000±0.000	1.000±0.000	–
Hermes–FedCM-style adapted	70.591±2.565	0.587±0.014	6.212±0.000	1.000±0.000	1.000±0.000	7.144±0.070
Adaptive-DP-spending adapted	70.591±2.565	0.587±0.014	6.199±0.000	0.000±0.000	1.000±0.000	7.113±0.056
BP-DR	70.551±2.564	0.587±0.014	6.205±0.000	1.000±0.000	1.000±0.000	7.325±0.049

Note: All values are reported as mean ± standard deviation over ten independent evaluation seeds. Adj. U, Reg. count, Bgt. ratio, and Ovhd. denote migration-adjusted utility, accepted regrouping count, final budget ratio, and average decision overhead per decision window, respectively. The overhead of Static grouping is omitted because it does not perform dynamic regrouping decisions.

## Data Availability

The publicly available Alibaba Cluster Trace 2018 was used to construct the workload sequences, and the publicly available FLamby Fed-Heart-Disease benchmark was used for the end-to-end federated training validation. The source datasets are available from the original repositories cited in this article. The processed workload sequences, simulation outputs, and experimental results generated during the current study are available from the corresponding author upon reasonable request.

## Supplementary Materials

The following supporting information can be downloaded at: https://www.mdpi.com/article/10.3390/e28091000/s1, Supplementary Files S1–S7: Supplementary Statistical Analysis for BP-DR, including 95% confidence-interval analyses for the Main Comparison, Workload Drift Robustness, Migration and Churn Stress, Candidate Ranking Behavior Analysis, Privacy-Budget Scarcity Evaluation, Scale Sensitivity, and End-to-End Federated Training Validation.
